# MAVS Protein Is Attenuated by Rotavirus Nonstructural Protein 1

**DOI:** 10.1371/journal.pone.0092126

**Published:** 2014-03-18

**Authors:** Satabdi Nandi, Shampa Chanda, Parikshit Bagchi, Mukti Kant Nayak, Rahul Bhowmick, Mamta Chawla-Sarkar

**Affiliations:** Division of Virology, National Institute of Cholera and Enteric Diseases, Beliaghata, Kolkata, West Bengal, India; Geisel School of Medicine at Dartmouth, United States of America

## Abstract

Rotavirus is the single, most important agent of infantile gastroenteritis in many animal species, including humans. In developing countries, rotavirus infection attributes approximately 500,000 deaths annually. Like other viruses it establishes an intimate and complex interaction with the host cell to counteract the antiviral responses elicited by the cell. Among various pattern recognition receptors (PAMPs) of the host, the cytosolic RNA helicases interact with viral RNA to activate the Mitochondrial Antiviral Signaling protein (MAVS), which regulates cellular interferon response. With an aim to identify the role of different PAMPs in rotavirus infected cell, MAVS was found to degrade in a time dependent and strain independent manner. Rotavirus non-structural protein 1 (NSP1) which is a known IFN antagonist, interacted with MAVS and degraded it in a strain independent manner, resulting in a complete loss of RNA sensing machinery in the infected cell. To best of our knowledge, this is the first report on NSP1 functionality where a signaling protein is targeted unanimously in all strains. In addition NSP1 inhibited the formation of detergent resistant MAVS aggregates, thereby averting the antiviral signaling cascade. The present study highlights the multifunctional role of rotavirus NSP1 and reinforces the fact that the virus orchestrates the cellular antiviral response to its own benefit by various back up strategies.

## Introduction

In response to viral infection there are several pattern recognition receptors (PRRs) such as the Toll-like receptor (TLR), Nod-like receptor (NLR), RIG-I-like receptor (RLR) along with the DNA sensors, which plays significant role in activation of cellular innate immune response [1]. Among RLRs, Retinoic Acid-Inducible Gene I (RIG-I) and Melanoma Differentiation-Associated protein 5 (MDA-5) are the cytosolic receptors which discriminate between various classes of RNA and DNA viruses in order to activate interferons (IFNs). RNA viruses can be sensed by MDA-5 (Picornaviruses), by RIG-I (Hepatitis C virus, Influenza virus, Newcastle disease virus, Sendai virus, Rabies virus, Reovirus, Vesicular stomatitis virus and Japanese encephalitis virus), or by both RIG-I and MDA-5 (Dengue and West Nile virus) [2], [3]. In addition, RIG-I recognizes dsRNA and 5′-triphosphate moiety, whereas the length of dsRNA determines the utilization of RIG-I and MDA-5 for recognition [4]. After binding with viral RNA, caspase activation and recruitment domain (CARD) of RIG-I and MDA-5 interacts with the CARD domain of a common adaptor protein; mitochondrial antiviral signaling protein (MAVS: also known as IPS-1/VISA/Cardif). This interaction leads to the formation of prion-like aggregates of MAVS CARD domain which signals IKKs and TBK1 for the activation of IRF3 and NF-κB pathways [5]. A highly synchronized activation of NF-κB and IRF-3 pathways lead to the assembly of an activating complex comprising various proteins that drive expression of IFN-β and other IFN mediated antiviral immunity [6]–[10]. In order to counteract this antiviral milieu, viruses have developed various strategies to inhibit the IFN-β secretion. Interestingly in different classes of virus, the MAVS protein is abrogated from functioning in order to handicap the primary innate immune response. In hepatitis B, hepatitis C and Coxsackievirus B virus, MAVS is cleaved from its mitochondrial membrane location making it ineffective for downstream signaling [11]–[14]. The PB1-F2 protein inhibits MAVS-mediated IFN synthesis by decreasing the mitochondrial membrane potential during influenza virus infection [15], [16]. During rotavirus (RV) infection, RIG-I/MDA-5-MAVS pathway leads to the up regulation of type I IFNs rather than TLR3/TRIF or PKR pathway [17]. As the virus enters in the host cell, that activation of the antiviral response by RV is dependent on MAVS/IPS-1 and IRF3 involving both RIG-I and MDA-5, however IFN-β secretion during RV infection is regulated by PKR (Protein kinase R) phosphorylation [18], [19]. The central role of MAVS protein during RV infection was shown by Sen *et al* where both transcriptional responses and IFN-β secretion were completely abrogated in MAVS^−/−^ MEFs (mouse embryonic fibroblasts) [18].

Rotaviruses are members of Reoviridae family and are the single most important etiologic agent of severe infantile (<5 years) non-bacterial diarrhoea in humans worldwide [20]. It is a non-enveloped icosahedral virus with 11 double stranded RNA segments. Each RNA segment encodes a functional protein except segment 11 which encodes two nonstructural proteins in +1 open reading frame (ORF) [21]. Therefore the virus encodes six structural (VP1-4, VP6-7) and six non structural proteins (NSP1-6) [20]. Non-structural protein 1 (NSP1) of RV is a 55 KD protein which plays a vital role in antagonizing the IFN immune response [22]-[26], [44]. NSP1 is also found to activate PI3K/AKT mediated anti-apoptotic pathway [27] through its ability to bind p85 subunit of PI3K for activation of AKT [28], resulting in efficient virus infection and replication. In addition, NSP1 has shown to downregulate p53 and TRAF2 (TNF receptor associated factor 2) proteins [29], [30]. Except RIG-I, NSP1 mediated degradation of the above said proteins are proteasome dependent. Thus, there are many circumstantial evidences suggesting a putative ubiquitin ligase property of NSP1 [31]. The amino terminus of NSP1 forms one or two zinc fingers, which contains a highly putative RING-E3 ubiquitin ligase domain [32]. The C-terminal domain of NSP1 is involved in IRF3 binding [33]. It was shown by Barro *et al* that wild-type NSP1, not the C-truncated form, is an antagonist of the IFN-signaling pathway [24]. It was also shown that RV NSP1 mediates degradation of IFN regulatory factors through targeting of the dimerization domain [34].

Previous studies have suggested the role of RIG-I/MDA5-MAVS signaling during RV infection. The critical role of MAVS in activating early antiviral transcriptional responses is validated in MAVS^−/−^ MEFs [17], [18]. Therefore the aim of the study was to know whether RV modulates RIG-I/MDA-5-MAVS pathway by directly affecting this protein. NSP1 has been shown to degrade RIG-I [26] but in absence of RIG-I, MDA5 can complement it and activate IFN through MAVS. Herein, results revealed that RV protein NSP1 also down regulates the adapter protein MAVS during RV infection when host PRR mediated IFN-β activation is critical. Importantly it was found that the degradation was RV strain independent in nature unlike IRF3 degradation. Until now NSP1 from different RV strains were found to target different regulatory factors for antagonizing the IFN response, but this is the first report where NSP1 is found to target the central protein MAVS unanimously in a strain independent manner. Although, the down regulation of IRFs can also serve the same function, the following finding suggests a backup strategy undertaken by the same viral protein for efficient IFN down regulation, in case IRFs are not completely degraded. The study highlights the multistep control of host innate immunity by a viral protein.

## Materials and Methods

### Reagents and antibodies

Proteasomal inhibitor MG132 was purchased from Sigma–Aldrich (St. Louis, MO, USA) which was used at 20 μM/ml final concentration. Poly dA dT (deoxyadenylic-deoxythymidylic) and poly IC (inosinic-cytidylic acid) were purchased from Invivogen (San Diego, CA, USA). Both were transfected in cells at a final concentration of 2 μg/ml and 10 μg/ml respectively. Rabbit polyclonal antibodies against SA11 NSP1 (480–497 amino acids) and NSP3 (full length) were gifted by Professor Taniguchi, Department of Virology and Parasitology, Fujita Health University School of Medicine, Aichi, Japan. Antibody against MAVS (06-1096) was purchased from Millipore (Billerica, MA). TBK1 (sc-9085) and His probe (sc-803), were from SantaCruz Biotechnology (CA, USA). Antibodies against GAPDH (2118), β-Actin (4967), CoxIV (4844), IRF3 (4302), Phospho-IRF-3 Ser396 (4947), NF-κB (3017), Lamin A/C (2032), pIκbα (2859) and Iκbα (9242) were from Cell Signaling Technology. Antibody against FLAG epitope (SAB4200071) was from Sigma. All antibodies were used at 1∶1000 dilutions except NSP1 and NSP3 (1∶3000).

### Cell culture and virus infection

Human intestinal epithelial (HT29) and human embryonic kidney epithelial (HEK293) cell lines were cultured in Dulbecco′s modified Eagle′s medium (DMEM) supplemented with 10% fetal bovine serum and 1% antibiotic–antimycotic solution (Invitrogen, Carlsbad, CA). Cells were maintained in 5% CO2 at 37°C humidified incubator. Various rotaviral strains used for the study were propagated and titers were determined with MA104 cells. For infection, viruses were activated with acetylated trypsin (10 μg/ml) at 37°C for 30 min and added to the cells for adsorption (45 min), followed by washing with media to remove unbound virus. Infection was continued in fresh medium. Except for experiments with HEK293 cells, an MOI of 1 was used for MA104 or HT29 cells in the study. The time of virus removal was taken as 0 hour post infection (hpi) for all experiments. The end point in experiments was determined based on time required by virus to complete its replication in cells resulting in an >80% cytopathic effect. The end point for HT29 cells varied from 18 to 24 h.

### Plasmid construction

Vectors expressing human IRF3-5D, human TBK1 and pTATA-luc-4x-IRF-3 are generous gifts from Dr. Aldofo Garcia-Sastre ( Mount Sinai School of Medicine), Dr Harry Greenberg (Stanford University) and Dr Stephan Ludwig (University of Munster) respectively All primers used in the study for respective constructs are given in Table 1. Full length NSP1, NSP2, NSP3, NSP4, NSP5 and ubiquitin were cloned in pcDNA6 vector (Invitrogen) with C-terminal His-Tag as described previously [29]. To prepare pcDNA vectors containing the NSP1 ORFs of RV strains OSU (accession number D38153), DS-1 (accession number EF672578), Wa (accession number L18943), KU (accession number AB022769) and different truncated mutants of NSP1, viral RNA was extracted from infected cells by using TRIzol LS reagent (Invitrogen). Gene 5 cDNAs were prepared from the RNA by reverse transcription (RT)-PCR followed by PCR with respective primer sets. Full length MAVS (accession number BC044952), region specific mutants of MAVS were cloned in pFLAG-CMV (Sigma, MO) vector using specific primers.

**Table 1:** List of primers designed and used in the study.

NAME	SEQUENCE	VECTOR
SA11 H96 F KpnI	5'GGT ACC ATG GCT ACT TTT AAA GAT GCA TGC TTT CAT	pCDNA6B
SA11 H96 R XhoI	5'CTC GAG TTC TCA TTG TCA TCT TCT GAG TTG GAG A	pCDNA6B
OSU F KpnI	5'GGT ACC ATG GCT ACT TTT AAG GAT GCT TGC TAT T	pCDNA6B
OSU R XhoI	5'CTC GAG TTT TCA ACA TCA GAT ATA CCG GAA TCA TAG	pCDNA6B
DS-1 F KpnI	5'GGT ACC ATG GCT ACT TTT AAA GAT GCT TGC TAT C	pCDNA6B
DS-1 R XhoI	5'CTC GAG TTT TCA ATA TCG GAT ATA CCT GAA TCA TGT	pCDNA6B
Wa F KpnI	5'GGT ACC ATG GCT ACT TTT AAA GAC GCT TGT TAT TAT	pCDNA6B
Wa R XhoI	5'CTC GAG TTT TCA ACA TCA GAT ATA CCA GAA TCA TAT	pCDNA6B
KU F KpnI	5'GGT ACC ATG GCT ACT TTT AAA GAT GCT TGT TAT C	pCDNA6B
OSU R XhoI	5'CTC GAG TTT TCA ACA TCA GAT ATG CCA GAA TCA TC	pCDNA6B
NSP1-N-100 R XhoI	5'CTC GAG TTA ATT GGA TGT TTC ACA GTT CTA AGC	pCDNA6B
NSP1-C-395 F KpnI	5'GGT ACC ATG ACC AAA GAC AAA TTA CAG TGT ATC	pCDNA6B
NSP1-del IRF3 BD Xho1	5'TCT ACT CGA GCG CCA TTT ACA GTA CTG GAT ATC G	pCDNA6B
MAVS F EcoRV	5'G ATA TCA TAT GCC GTT TGC TGA AGA CAA GAC C	pCMVFLAG6b
MAVS R KpnI	5'G GTA CCA CTA GTG CAG ACG CCG CCG GTA CAG	pCMVFLAG6b
MAVS-CARD R KpnI	5'G GTA CCC TAC GAG GTC CGA GGC TGG TAG CTC TC	pCMVFLAG6b
MAVS-CARD-TM F	5'CCA GGG CCC CCC GGG GCT CTG TGG CTC	
MAVS-CARD-TM R	5'GGA GCC ACA GAG CCC CGG GGG GCC CTG GCC TCT CAG	
Pro-MAVS F EcoRV	5'G ATA TCA GAC CGT CCC CCA GAC CCA CTG	pCMVFLAG6b

### Quantitative real-time RT-PCR

Total RNA was isolated using TRIzol (Invitrogen, Grand Island, USA) according to the manufacturer's instructions. cDNA was prepared from 1–2 μg of RNA using the Superscript II reverse transcriptase (Invitrogen) with random hexamer primers. Real-time PCR reactions (50°C for 2min, 95°C for 10 min, followed by 40 cycles of 95°C for 15 s and 60°C for 30 s and 72°C for 10 min) were performed in triplicate using SYBR Green (Applied Biosystems, Foster City, CA, USA) with primers specific for GAPDH (F-5′-AATCCCATCACCATCTTCCAG-3′ and R-5′- AAATGAGCCCCAGCCTTC-3′), nsp4 (F-5′-GTGCAAACGACAGGTGAAATAG-3′ and R-5′-AGTCACTTCTCTTGGTTCATAAGG -3′), ISG56 (F-5′-TGGGCCTTGCTGAAGT-3′ and R-5′-GGCCCATCCTTCCTCA-3′), MAVS TaqMan probe ID, IFN-β TaqMan probe ID Hs01077958_s1 were used. Relative gene expressions were normalized to GAPDH using the formula 2^−ΔΔCT^ (ΔΔCT = ΔC_Tsample_-ΔC_T untreated control_).

### Gel electrophoresis and immunoblot analyses

Whole cell lysates was prepared with Totex buffer (20 mM Hepes at pH 7.9, 0.35 M NaCl, 20% glycerol, 1% NP-40, 1 mM MgCl_2_, 0.5 mM EDTA, 0.1 mM EGTA, 50 mM NaF and 0.3 mM Na_3_VO_4_) containing mixture of protease and phosphatase inhibitors (Sigma, St. Louis, MO), Samples were incubated in protein sample buffer (final concentration: 50 mM Tris, pH 6.8, 1% SDS, 10% glycerol, 1% β-mercaptoethanol, and 0.01% bromphenol blue) for 30 min at either 4 °C or, alternatively, boiled for 5 min before running SDS-PAGE at room temperature followed by immunoblotting with specific antibodies. Primary antibodies were identified with HRP conjugated secondary antibody (Pierce, Rockford, IL) and chemiluminescent substrate (Millipore, Billerica, MA). To confirm protein loading blots were reprobed with GAPDH. The immunoblots shown are representative of three independent experiments. Blots were scanned and quantitated using GelDoc XR system and Quantity One software version 4.6.3 (BioRad, Hercules, CA).

### Nuclear protein extraction

Nuclear and cytosolic protein extracts were prepared with ProteoJET Cytoplasmic and Nuclear Protein Extraction Kit (Fermentas Life Science, USA) for NFκB p52 and IRF3 translocation studies.

### Immunoprecipitation

Cell lysate from infected HT29 cells or transfected HEK293 cells were clarified followed by incubation with specific antibodies overnight at 4°C and with protein A-Sepharose beads (GE Healthcare, Sweden), for 4h. Beads were washed 5 times with 1 ml wash buffer (200 mM Tris pH-8.0, 100 mM NaCl and 0.5% NP-40) and bound proteins were eluted by boiling for 5 min with SDS sample buffer before separation on 12% SDS-PAGE gels followed by immunoblotting with specific antibodies.

### Transient transfections and reporter gene assays

HEK293 cells were transfected with Lipofectamine 2000 (Invitrogen) according to the manufacturer's instructions. IRF3 luciferase reporter gene assays were performed by transfection of constructs pCMV-MAVS, pTATA-luc-4x-IRF-3 (0.15 mg per 24-well), which contains the luciferase reporter gene under the control of four copies of the IRF-3 binding PRDI/III motif of the IFN-β promoter [35] along with presence or absence of pcD-NSP1. For dual luciferase NFκB reporter assay, HEK293 cells were cotransfected with NFκB–luc (containing IL8 promoter), pRL-TK, pCMV-MAVS and pcD-NSP1 or pcDNA. 24 hour post transfection, the luciferase activity was measured according to the manufacturer’s protocol (Promega) using a luminometer (Varioskan multimode reader; Thermo Fisher).

### SDD-AGE (semidenaturing detergent agarose gel electrophoresis)

Semidenaturing detergent agarose gel electrophoresis (SDD-AGE) was performed according to the protocol followed by Hou *et al* [5]. In brief, crude mitochondria from HEK293 cells were isolated in Buffer A (10 mM Tris-HCl [pH 7.5], 10 mM KCl, 1.5 mM MgCl2, 0.25 M D-mannitol, and Roche EDTA-free protease inhibitor cocktail) and then resuspended in 1x sample buffer without β-mercaptoethanol (0.5× TBE, 10% glycerol, 2% SDS, and 0.0025% bromophenol blue). Mitochondrial pellets were run on 1.5% agarose gel (Bio-Rad) in the running buffer (1 x TBE and 0.1% SDS) for 3–4 hours with a constant voltage of 50 V at 4°C. The proteins were transferred to nitrocellulose membrane by capillary method for 6 hours (RT) followed by immunoblotting with MAVS Antibody.

### Statistical analysis

Data are expressed as mean±standard deviations of at least three independent experiments (n≥3). Results from all studies were compared with unpaired two-tailed Student's t test and p<0.05 was considered statistically significant.

## Results

### Degradation of MAVS during Rotavirus infection

**Figure 1:**
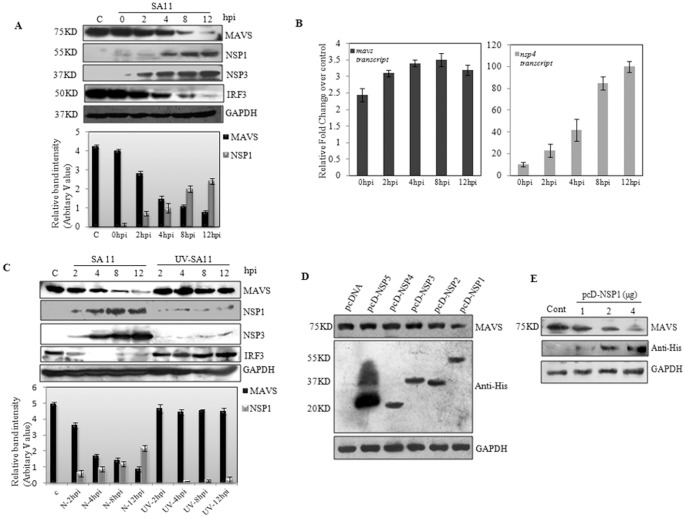
MAVS is degraded during rotavirus infection. **A)** HT29 cells were infected with RV strain SA11 (1 M.O.I.) and cell lysates were prepared at indicated time points. Proteins were separated on 12.5% SDS-PAGE and immunoblotted using MAVS Ab. Membranes were reprobed with NSP1 NSP3, IRF3, and GAPDH antibodies as internal control. Band intensities of MAVS and NSP1 were normalized to the loading control GAPDH and expressed as a percentage of the protein in mock infected cells. **B)** HT29 cells were infected with SA11 at (1 M.O.I.) for indicated time points. RNA was isolated and the *nsp4* and *mavs* transcripts were analyzed by qRT-PCR. Fold changes were obtained by normalizing relative gene expressions to *gapdh* using the formula 2^−ΔΔCT^ (ΔΔC_T_ = ΔC_TSample_-ΔC_TUntreated control_). **C)** Expression of MAVS in HT29 cells infected with normal or psoralane-UV-irradiated replication deficient RV SA11 simian strain. Band intensities of MAVS and NSP1 were expressed in percentage. **D)** HEK293 cells were transfected with pcDNA constructs of NSP5, NSP4, NSP3, NSP2 and NSP1. The expression of MAVS protein was analyzed and expression of viral NSPs were confirmed by immunoblotting using His Ab. **E)** Dose-dependent degradation of MAVS protein in total cell extracts of HEK293 cells transfected with increasing concentration of pcD-NSP1 for 24 hours. The data represent the means ± the standard deviations (SD) of three independent experiments.

The expression of MAVS was analysed during the time course of RV infection in HT 29 cells infected with SA11 (1 M.O.I.) followed by immunoblot analysis of the cell lysates with MAVS specific antibody. Immunoblotting revealed degradation of MAVS protein after infection in a time dependent manner (Figure 1A). To assess whether RV mediated regulation of MAVS is transcriptional or post transcriptional, transcript were quantitated by realtime PCR in SA11 infected HT29 cells (0–12 hpi). There was no significant change of MAVS mRNA (Figure 1B) and thus the degradation of MAVS after infection was confirmed as a post-transcriptional phenomenon. To elucidate whether rotaviral entry or replication is responsible for MAVS degradation, HT 29 cells were infected with either normal or psoralane-UV-irradiated replication deficient virus [36]. The extent of virus replication inability was confirmed by estimating viral titer and quantitating viral transcript by qRT-PCR (Figure S1). As shown in Figure 1C, in spite of being structurally and immunologically competent, UV treated virus could not degrade MAVS suggesting that viral replication and synthesis of viral nonstructural proteins plays a role in the process. Furthermore, when HEK293 cells were transfected with constructs encoding NSPs (NSP1-NSP5) followed by immunoblotting after 24 hours. MAVS degradation was observed only in NSP1 over expressing cells (Figure 1D). To confirm the loss of MAVS in presence of NSP1, total cell lysates were prepared, 24h after transfection of HEK293 cells with increasing concentration of pcD-NSP1. Immunoblotting revealed degradation of MAVS with increasing concentration of pcD-NSP1 (Figure 1E).

### Rotavirus strain independent degradation of MAVS

**Figure 2:**
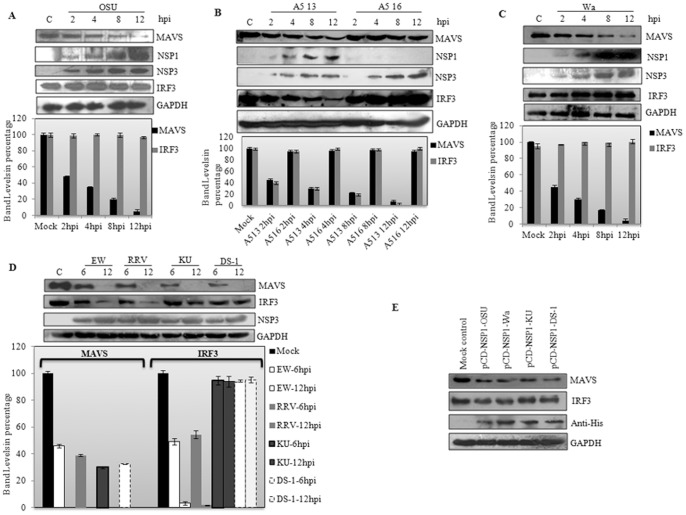
Degradation of MAVS in RV infected HT29 cells and NSP1 transfected HEK293 cells. **A, B, C and D)** HT29 cells were infected with the indicated strains of RV (1 M.O.I.) and harvested at various time points. Total cellular protein was harvested and separated by SDS-PAGE. Immunoblot analyses were performed with antibodies to the cellular proteins MAVS, IRF3, GAPDH and the viral proteins NSP1 and NSP3. Porcine OSU, bovine A5-13, A5-16 and human Wa were harvested at 2, 4, 8 and 12 hpi. Murine EW, rhesus RRV, human KU and DS-1 were lysed at 6 and 12hpi. Band intensities of MAVS and IRF3 were normalized to the loading control GAPDH and were expressed as a percentage of the protein in mock infected cells. The data represent the means ± SD of three independent experiments. **E)** Effects of NSP1 proteins of different RV strains on human MAVS. HEK293 cells were transfected with vectors encoding indicated RV NSP1 proteins and harvested at 24 h p.i. Proteins in cell lysates were resolved by SDS-PAGE and analyzed by immunoblotting for MAVS, IRF3 and Anti-His antibody.

Previous studies have shown diverse IFN antagonist activities mediated by NSP1 of different RV strains [37].Thus, MAVS degradation was analyzed in HT29 cells following infection with several common laboratory strains of RV namely porcine OSU, rhesus RRV, bovine A5-13 and A5-16, human Wa, KU and DS-1 (1 M.O.I.). Cell lysates were immunoblotted with MAVS specific antibody. As an internal control IRF3 degradation was also analyzed in same blots. Blots were reprobed with NSP3 antibody to confirm the infection and replication of RV strains in HT29 cells. As shown in Figure 2, except for A5-16 where a truncated NSP1 is synthesized, infection with all other strains abrogated MAVS by almost 50% compared to the mock infected control, whereas IRF3 degradation was observed only in A5-13, EW and RRV strains. No IRF3 degradation was observed in OSU, Wa, KU or DS-1 infected cells. This suggests that MAVS degradation is independent of RV strain as well as its ability to degrade IRF3.

Furthermore, *nsp1* gene from four RV strains was cloned in pcDNA vector and transiently transfected in HEK293 cells. After 24 hours, cell lysates were immunoblotted for MAVS and IRF3 expression. Consistent with the previous results with RV strains, degradation of MAVS was observed in cells expressing NSP1 whereas no significant effect on IRF3 was observed. Overall results suggested that MAVS is an important target during RV infection.

### Proteasome inhibitor interferes with MAVS degradation

**Figure 3:**
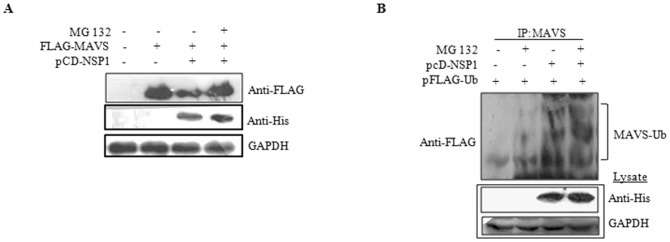
Proteosome-mediated MAVS degradation. **A)** NSP1 induced MAVS degradation is prevented in presence of MG132. HEK293 cells were either transfected with FLAG-MAVS or co transfected with pFLAG-MAVS and pcD-NSP1 in presence or absence of 20 μM MG132 followed by immunoblot analysis with anti-FLAG, anti-GAPDH and anti-His. **B)** NSP1 induces ubiquitinylation of MAVS. HEK293 cells were transfected with expression vector encoding FLAG-tagged ubiquitin with presence or absence of pcD-NSP1. Cells were grown in DMEM containing MG132 (20 μM) for 6 h. Anti-MAVS immunoprecipitates were analyzed by immunoblotting His-tagged ubiquitin with anti-His Ab. Whole-cell lysates were subjected to immunoblotting with anti-His and anti-GAPDH was used as an equal loading control. Figure also showed that MG132 affects MAVS degradation but not ubiquitinylation.

To determine whether the NSP1-dependent loss of MAVS in RV-infected cells occurred via the proteasome pathway, HEK293 cells were treated with MG132 (20 μM for 6 h) following co-transfection of pFLAG-MAVS and pcD-NSP1. As shown in Figure 3A, MAVS protein expression was significantly restored in presence of MG132. To further elucidate the mechanism, *in vitro* ubiquitination assay was performed where HEK293 cells were cotransfected with plasmids expressing pFLAG-NSP1 together with pFLAG-ubiquitin in the presence or absence of proteasome inhibitor MG132 (20 μM for 6 h), followed by immunoprecipitation using anti-MAVS Ab. Immunoblot analysis using anti-FLAG Ab revealed that full length NSP1 resulted in ubiquitination of MAVS. These results indicate that NSP1 has a role in MAVS ubiquitinylation and its proteasome-mediated degradation.

### Rotavirus NSP1 inhibits MAVS mediated interferon beta activation irrespective of IRF3 degradation

**Figure 4:**
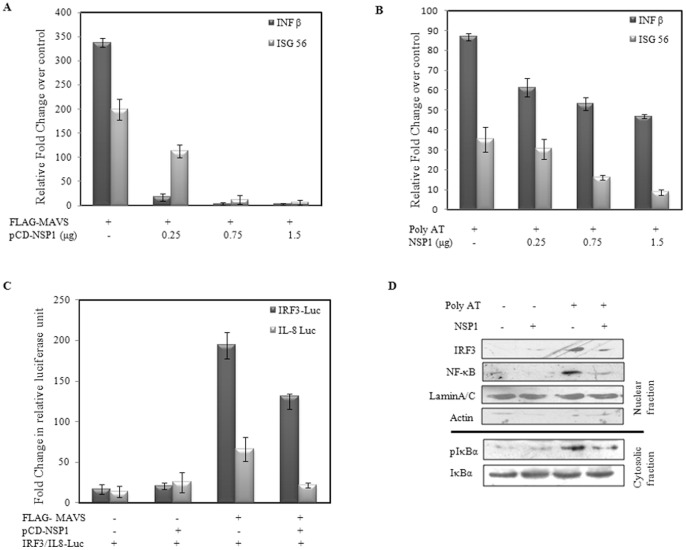
NSP1 inhibits, MAVS mediated IFN-β induction. **A & B)** Fold change of IFN-β and ISG 56 were calculated in presence or absence of NSP1 in MAVS overexpressing, or poly AT stimulated cells. RNA was extracted from transfected HEK293 and qRT-PCR was performed with SYBR green Mastermix. GAPDH was used as reference gene. Data presented as fold change (based on 2^−ΔΔCt^ values) relative to mock transfected control cells (mean ± SD; n = 3). **C)** Relative increase in IRF3 and NF-κB promoter activity was measured in cells transfected the pLuc-4xIRF3 or pLuc-IL8 luciferase reporter vectors, pRL-TK as loading control in absence or presence of pcD-NSP1. Luciferase activities were determined using the Dual Luciferase Reporter Assay Kit (Promega, Madison, WI). The data are presented as the fold change in luciferase units (mean ± SD; n = 3, P<0.05) relative to mock control and was normalized with the Renilla luciferase activity. **D)** Cytosolic and nuclear proteins were isolated from HEK293 cells with ProteoJET Cytoplasmic and Nuclear Protein Extraction Kit. Cytoslic fractions were analyzed for pIκBα and nuclear fraction aliquots were immunoblotted with IRF3, NFκB, LaminA/C and actin antibodies.

To determine the downstream role of MAVS degradation mediated by NSP1, we co-expressed pFLAG-MAVS and pcD-NSP1 constructs in HEK293 cells and qRT-PCR was performed. In MAVS expressing cells, >300 fold induction of IFN-β and 200 fold induction of ISG56 transcripts was observed (Figure 4A). However when NSP1 was co-expressed, significant downregulation of both IFN-β and ISG56 was observed in a dose dependent manner (Figure 4A). To mimic virus induced activation of innate immune response in cells, the cells were transfected with poly dA dT which transcribes into dsRNA with a 5′-triphosphate moiety (5′pppdsRNA) and is indirectly sensed by RIG-I [1, 18 and 19]. In HEK293 cells, poly dA dT transfection resulted in significant increase in IFN-β (>80 fold) and ISG56 (>30 fold) transcript compared to control (Figure 4B), which was inhibited in presence of increasing concentration of NSP1 (Figure 4B). Similar results were obtained with Poly IC, a synthetic dsRNA analogue (data not shown).

IRF3 and NFκB luciferase assays were performed to study the functional role of NSP1 in MAVS dependent IRF3 and NFκB activation. For IRF3 reporter assay, HEK293 cells were cotransfected with IRF3 luciferase reporter vector, pCMV-MAVS in presence or absence of pcD-NSP1. For dual luciferase NFκB reporter assay, HEK293 cells were cotransfected with NFκB–luc (containing IL8 promoter), pRL-TK, pCMV-MAVS and pcD-NSP1 or pcDNA. The results revealed more than 50 fold reduction in both IRF3 and NFκB activation in presence of NSP1 as evident in Figure 4C. For confirmation, the nuclear fractions were isolated from poly AT transfected (12h) HEK293 cells expressing either pcD-NSP1 or empty vector control. Consistent with the qRT-PCR data, in cells expressing NSP1, significantly less nuclear translocation of IRF3 and NFκB was observed. The levels of pIκBα were checked in the cytosolic fraction which revealed lower phosphorylation in presence of NSP1 (Figure 4D).

**Figure 5:**
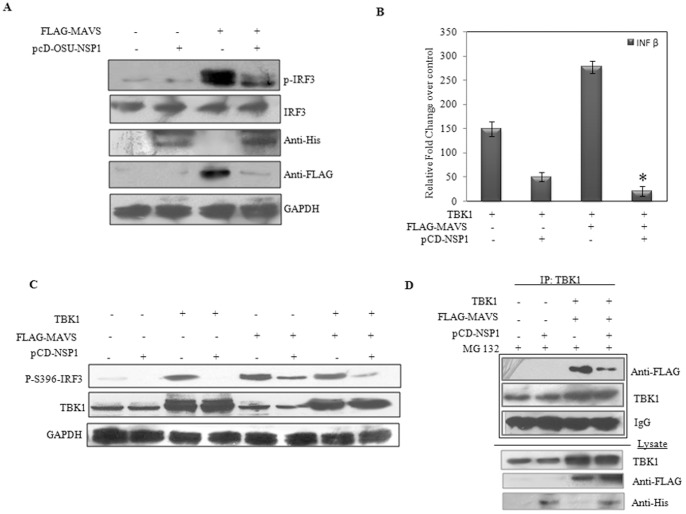
NSP1 inhibits IFN-β induction irrespective of IRF3 degradation. **A)** HEK293 cells were transfected with FLAG-MAVS and pcD-OSU-NSP1 vector in order to assess the MAVS mediated inhibition of IRF3 phosphorylation. Cell lysates were analyzed for pIRF3, IRF3, Anti-His, Anti-FLAG and GAPDH specific antibodies. **B)** Fold change of IFN-β transcripts was assessed in cells overexpressing human TBK1 and pFLAG-MAVS vectors, in presence or absence of pcD-NSP1. The data shown are means ± the SD (n = 3). * Significantly different in comparison to human TBK1 and NSP1 transfected and pFLAG-MAVS untransfected condition. P<0.05 **C)** Activation of IRF3 was assessed in absence or presence of pcD-NSP1 in cells transfected with TBK1 and FLAG-MAVS. **D)** Association of MAVS with TBK1 was studied by Co-IP in presence or absence of pcD-NSP1 in cells overexpressing TBK1 and MAVS. The MAVS degradation was controlled by proteosomal inhibitor MG132 Results reveal reduced interaction between MAVS-TBK1 in presence of NSP1.

As NSP1 is already known to downregulate both IRF-3 and NF-kB activation [24], it was necessary to assess whether degradation of MAVS has actually any direct effect on IFN induction. Since previous report has shown inability of OSU NSP1 to downregulate IRF3 [31], the NSP1 construct from OSU strain was used. pFLAG-MAVS vector was co-transfected with pcD-NSP1-OSU and IRF3 phosphorylation was checked. As shown in Figure 5A, pcD-NSP1-OSU was able to inhibit IRF3 phosphorylation, all though there was no degradation of IRF3. When the membranes were reprobed with MAVS antibody there was loss of MAVS protein in cells expressing OSU-NSP1. These results were consistent with our previous results in Figure 2A where MAVS was degraded even in OSU infected cells. Thus, NSP1 can modulate IFN induction by MAVS degradation irrespective of its IRF3 degrading property. Furthermore, TBK1 and IRF3-5D, a phosphomimetic form of IRF3 were overexpressed, along with NSP1 in presence and absence of MAVS to analyze the induction of IFN-β transcripts. As overexpression of TBK1 or IRF3-5D results in constitutively phosphorylated IRF3, our aim was to analyze effect of NSP1 on MAVS in presence of constitutively active IRF3. As shown in Figure 5B, in presence of both NSP1 and MAVS, the IFN-β levels induced by TBK1 overexpression, were significantly inhibited compared to only pcD-NSP1 transfected cells. Similar results were achieved when IRF3 phosphorylation was measured by western blotting. As shown in Figure 5C, IRF3 phosphorylation was inhibited more in cells overexpressing NSP1 along with MAVS and TBK1 compared to only TBK1. Comparable results were achieved with overexpression of IRF3-5D (Figure S2). In addition, interaction between TBK1 and MAVS was analysed in presence and absence of NSP1 by Co-IP. As MAVS is reported to interact with TBK1 protein for its downstream functioning, it was hypothesized that degradation of MAVS could directly affect its interaction with TBK1. HEK293 cells were transfected with pFLAG-MAVS and pcD-NSP1 in the presence of MG132. Experiments confirmed reduced interaction of MAVS-TBK1 in presence of NSP1 (Figure 5D). Thus, ubiquitinylation of MAVS protein hampers its proper functioning as an adapter molecule. Overall, results confirmed that even in presence of activated IRF3 there is considerable downregulation of IFN response which is due to degradation of MAVS protein during RV infection.

### NSP1 interacts with MAVS protein

**Figure 6:**
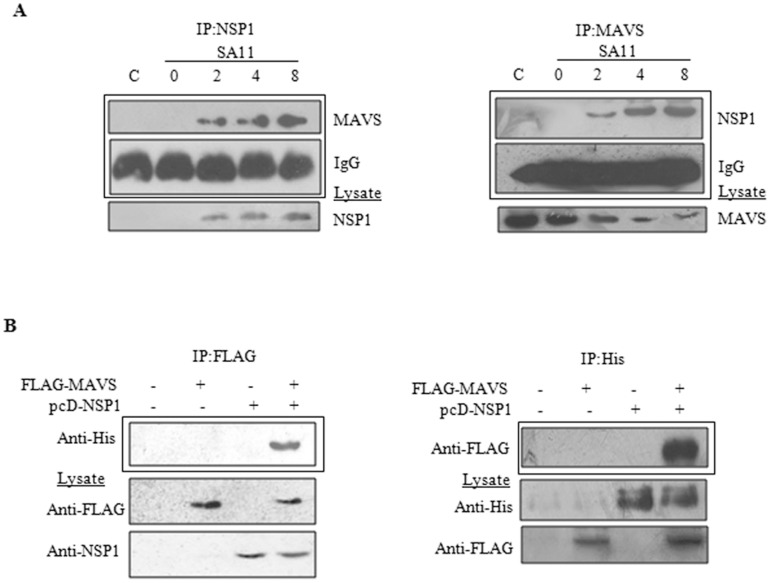
NSP1 interacts with MAVS protein during infection and transfection. **A)** NSP1 interacts with MAVS during SA11 infection. HT29 cells were infected with SA11 for increasing time points and cell extracts were immunoprecipitated with either NSP1 or MAVS antibody followed by immunoblotting with reciprocal antibody. Whole cell lysates were immunoblotted with anti-MAVS and anti-NSP1 antibody. CO-IP revealed positive interaction between NSP1 and MAVS. **B)** HEK293 cells were co-transfected with pFLAG-MAVS and pcD-NSP1. After 24 hours, cell extracts were immunoprecipitated using FLAG Ab or His Ab followed by immunoblotting by reciprocal antibodies. NSP1 was found to co-immunoprecipitate with MAVS in absence of other viral protein.

Role of NSP1 in MAVS degradation was confirmed, but whether NSP1 directly interacted with MAVS to cause degradation or it was an indirect effect was not clear. To analyze, the cells were infected with SA11 for 0-8 hours and co-immunoprecipitation was done with the NSP1 antibody followed by immunoblotting with MAVS. As shown in Figure 6A, interaction between NSP1-MAVS was observed as early as 2hpi which gradually increased until 8 hpi. The interaction of NSP1 with MAVS was confirmed by reciprocal co-IP experiments as shown in Fgure 6B. To rule out role of any other rotaviral protein during this interaction, pcD-NSP1 and FLAG-MAVS were co-transfected in HEK293 cells. Cells were lysed 24 hours post-transfection, and CO-IP (Pierce IP kit) was performed with FLAG or His antibodies targeting MAVS or NSP1 respectively. Immunoblotting with reciprocal antibodies revealed that there is a significant interaction between NSP1 and MAVS protein (Figure 6C).

**Figure 7:**
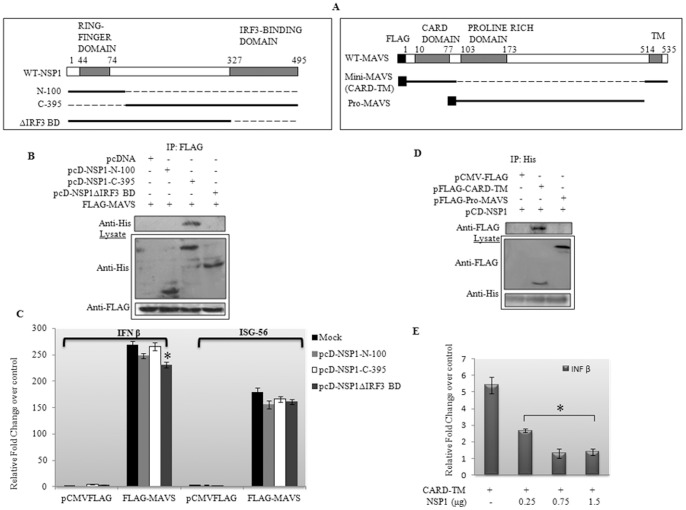
Domains of NSP1 and MAVS responsible for the interaction. **A)** Diagrammatic representation of mutants of NSP1 and MAVS used in the study. For NSP1 there were 3 mutants; pcD-NSP1-100 which comprised the putative ubiquitin ligase domain, pcD-NSP1-C-395 containing the remaining 395 amino acids of C-terminal and pcD-NSP1ΔIRF3BD comprising the N-terminal 327 amino acids. For MAVS, two constructs were generated, one comprising the CARD and TM domain (pFLAG-CARD-TM) and other vector encoding the intermediate proline rich domain of MAVS (pFLAG-Pro-MAVS). **B)** To map the domain of NSP1 responsible for this interaction, HEK293 cells were co-transfected with the NSP1 mutants and pFLAG-MAVS. Pull down was performed with anti FLAG Ab which revealed a positive interaction between pcD-NSP1-C-395 and MAVS. Membrane was probed with Anti-FLAG Ab. **C)** Relative fold change over control was measured for IFN-β and ISG 56 gene transcripts in cells co-expressing full length NSP1-N-100, NSP1-C-395 and NSP1ΔIRF3BD with MAVS. The data shown are means ± the SD (n = 3). * Significantly different in comparison to mock transfected condition. P<0.05.**D)** To identify the domain of MAVS protein responsible for the interaction, two construct pFLAG-CARD-TM (Mini MAVS) and pFLAG Pro-MAVS were co-transfected with pcD-NSP1 and pulled down with anti-His Ab. CARD-TM domain of MAVS interacts with NSP1. **E)** Relative fold change in IFN-β gene transcript in cells expressing mini-MAVS in presence or absence of NSP1 with respect to control was measured by real-time PCR. The data shown are means ± the SD (n = 3). * Significantly different in comparison to pcD-NSP1 untransfected condition. P<0.05.

In order to map the domains responsible for this interaction, deletion mutants of NSP1 and MAVS were constructed. pcD-NSP1-N100 (N terminal 100 amino acids including the putative the RING-E3 ubiquitin ligase domain), pcD-NSP1-C395 (C-terminal 101 395 amino acids) and pcD-NSP1ΔIRF3BD (NSP1 lacking IRF3 binding domain) (Figure 7A). Two construct were generated for MAVS, Mini MAVS, comprising of only CARD domain followed by the TM domain and Pro-MAVS comprising the remaining proline rich domain. The CARD domain is responsible for the interaction of MAVS with upstream signaling proteins and the TM region is responsible for the mitochondrial translocation. Co- immunoprecipitation experiments carried out in cells expressing pcD-NSP1-N100 or pcD-NSP1-C395 or pcD-NSP1ΔIRF3BD, revealed strong interaction between C- terminal fragment (NSP1-C395) and MAVS protein (Figure 7B). Both pcD-NSP1-N100 and pcD-NSP1ΔIRF3BD did not interact with MAVS. To correlate Co-IP results with functionality, HEK293 cells were co-transfected with MAVS and either pcD-NSP1-N100 or pcD-NSP1-C395 or pcD-NSP1ΔIRF3BD and IFN-β and ISG56 transcripts were analysed by real-time PCR. In spite of interaction with MAVS, C-395 domain of NSP1 could not inhibit MAVS induced IFN-β (Figure 7C). Unlike FL-NSP1, pcD-NSP1-N100 and pcD-NSP1ΔIRF3BD constructs also could not inhibit MAVS induced IFN-β (Figure 7C).

In order to map the domain of MAVS responsible for its interaction with NSP1, Co-IP experiments were carried out in cells expressing pCD-NSP1 along with pFLAG-CARD-TM or Pro-MAVS. Co- immunoprecipitation experiment revealed that similar to full length MAVS, NSP1 can interact with mini MAVS (Figure 7D). Mini-MAVS has also been shown to induce IFN-β similarly to WT MAVS [13]. Thus, in order to assess whether this interaction has any effect on CARD-TM expression levels, HEK293 cells were co-transfected with pFLAG-CARD-TM along with increasing concentration of NSP1. Consistent with previous results with full length MAVS, NSP1 degraded Mini-MAVS (CARD-TM) as well as inhibited IFN-β transcript >50 fold (Figure 7E). Expression of Mini-MAVS and NSP1 in cells were confirmed by immunoblotting (Figure S3). Overall results suggest that CARD domain and TM domain of MAVS are sufficient to interact with NSP1 and inhibit IFN pathway, however, full length NSP1 is required for degradation of MAVS.

### Identification of MAVS aggregates and its inhibition by NSP1

**Figure 8:**
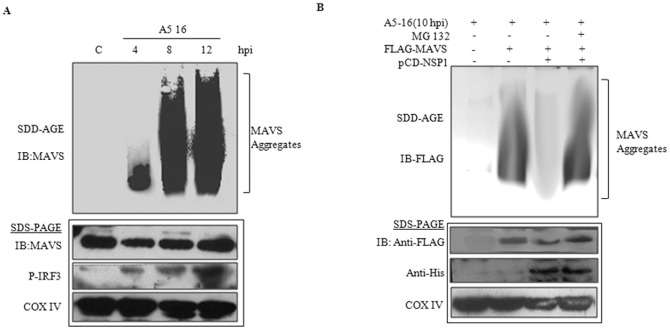
Formation of MAVS aggregates during Rotavirus infection. **A)** Crude mitochondrial extracts were prepared from HEK293 cells infected with A5-16 strain (3 M.O.I.) at increasing time points (4, 8 and 12). Extracts were analyzed by SDD-AGE to assess the MAVS aggregate formation. Results revealed MAVS aggregate formation from 4 hpi. **B)** Role of NSP1 on MAVS aggregation and ubiquitinylation was observed by overexpressing FLAG-MAVS in absence or presence of NSP1. Infection of A5-16 was used for inducing MAVS aggregation, as overexpression of MAVS alone is insufficient for inducing aggregate formation. Results show inhibition of MAVS aggregates in presence of NSP1, which gets restored following MG132 (20 μM) treatment. A fraction of SDD-AGE lysate was analyzed by SDS-PAGE followed by immunoblotting of MAVS, p-IRF3 and COX IV.

It has been reported that during viral infection MAVS forms large detergent resistant aggregates which activate IRF3 in cytosol [5].To analyze kinetics of MAVS aggregate formation during RV infection and role of NSP1 in this process, SDD-AGE (semidenaturing detergent agarose gel electrophoresis) was used as described previously [5]. A fraction of the SDD-AGE lysate was kept for running SDS-PAGE in order to analyze the expression of MAVS, phospho-IRF3 and COX IV as internal control. The NSP1 mutant RV strain A5-16 was used for virus infection, since it does not degrade cellular MAVS (Figure 2B). As shown in Figure 8A, it was noted that MAVS antibody could barely detect MAVS in uninfected control and 4 hpi infected cells on SDD-AGE, whereas MAVS expression was confirmed in the same samples following regular SDS-PAGE. Thus MAVS aggregates are formed as early as 4hpi in infected cells which co-relates with downstream signaling as shown by the phosphorylation of IRF3. In order to confirm the role of NSP1 on MAVS aggregate formation, HEK293 cells were transfected with pcD-NSP1 and FLAG-MAVS protein followed by infection with A5-16 (NSP1 mutant) strain, in presence of either MG132 or DMSO. Virus infection was done to induce MAVS aggregation as MAVS overexpression alone induces very low level of aggregates [5]. Membrane was immunoblotted with anti-FLAG antibody to avoid detection of cellular MAVS. In cells overexpressing only MAVS, A5-16 infection resulted in the formation of MAVS aggregates (Figure 8B-lane 2). In cells overexpressing both NSP1 and MAVS, no aggregates were observed (Figure 8B-lane 3), however in presence of MG132 aggregates were restored (Figure 8D-lane 4). Thus, degradation of MAVS by NSP1 not only affects its interaction with downstream molecules (TBK1) but also inhibit downstream aggregate formation on mitochondrial membrane in response to virus infection.

## Discussion

Host cellular response to virus infection involves the concomitant activation of parallel signaling pathways leading to the transcription of a plethora of cytokine genes, prominent among these are the genes encoding type-I IFN. It is the action of IFNs that interconnect the innate immune responses into the adaptive response, which act in an auto-, para- and endocrine manner designed to defend against viral infection [38]. Several viruses have evolved sophisticated mechanisms to evade the host innate immune response by directly interfering with the activation or downstream signaling events associated with PRR signal propagation [39]. As it is known that MAVS is a potent inducer of IFN-I responses [6], [40]–[42], therefore it was important to determine whether RV regulates this adapter protein during virus replication. Here it is shown that rotaviral NSP1 down regulates the adaptor protein MAVS by proteosomal degradation thereby creating an environment devoid of viral RNA sensing machinery.

NSP1 subverts the host innate immune response by inducing the degradation of factors necessary for IFN-β signaling. Until now NSP1 is reported to down regulate IRF3, IRF5, IRF7, β-TrCP and RIG I [23]–[26], [44]. In addition, recently NSP1 was found to inhibit STAT1 activation [43] and induce NSP1 mediated degradation of p53 and TRAF2 proteins [29], [30]. A detailed study have been done by Arnold *et al* on the role of NSP1 from different virus strains, in targeting IRFs for inhibiting IFN-β [37]. There it was revealed that NSP1 of RV strains OSU, KU, Wa and DS-1 were unable to degrade IRF3 but were still competent enough to inhibit IFN-β response. OSU was found to degrade β-TrCP whereas KU, Wa and DS-1 targeted IRF5 and IR7. The finding that MAVS protein is also degraded during rotaviral infection brings forth the fine-tuning strategies carried out by the virus under different conditions. Our study shows degradation of MAVS during infection in a RV strain independent fashion. Extent of MAVS degradation varies from one RV strain to another with respect to control but does not co-relate with levels of IRF3 (Figure 2D). In addition overexpression of NSP1 from OSU, KU, DS-1 or Wa strains also resulted in degradation of MAVS irrespective of their ability to degrade IRF3. Therefore the ability of an NSP1 protein to degrade one target protein does not necessarily mean that it can also degrade other protein. This clearly indicates the presence of various complementary inhibition mechanisms. Thus, MAVS is universally targeted by the RV strains, during the early steps of infection to escape IFN induced antiviral signaling.

Since the RV NSP1 of most strains is highly competent in degrading IRFs it was difficult to devise an assay where the sole effect of MAVS down regulation on IFN modulation could be studied. To overcome this OSU-NSP1 was overexpressed as it has been shown to be defective in IRF3 degradation [37]. Results confirm no effect on IRF3 in OSU-NSP1 expressing HEK293 cells but MAVS degradation and inhibition of MAVS induced IRF3 phosphorylation was observed suggesting that both mechanisms are independent (Figure 5A). When IFN-β transcripts were assessed, inhibition of MAVS induced IFN-β was also observed in presence of OSU-NSP1 (data not shown). This is consistent with the previous report where inhibition of IFN-β was found in OSU infected HT29 cells [37]. This was further confirmed when TBK1 was overexpressed along with MAVS and full length NSP1 (SA11). As expected NSP1 inhibited TBK1 induced IFN-β response to almost one-third compared to only TBK1. When MAVS was further co-expressed with TBK1, a huge induction (>250 fold) of IFN-β was observed which was significantly downregulated (>80%) in presence of NSP1 (Figure 5B), confirming the significance of MAVS degradation. Overall it can be postulated that MAVS degradation is a part of the whole IFN antagonizing property of NSP1.

NSP1 has a RING domain in the N terminal (1-82 amino acids) which performs the ubiquitinylation of specific proteins in a fashion similar to ubiquitin conjugating enzymes (E2). Previous reports suggest NSP1 induces degradation of IRFs and β-TrCP proteosomally except RIG-I, which is degraded by a different unknown mechanism. In our present study MAVS degradation was also found to be proteosome mediated, as it was rescued in presence of proteosomal inhibitor, MG132. To know whether NSP1 binds with MAVS for its degradation, co-immunoprecipitation was done both in cells infected with RV strain SA11 as well as in cell overexpressing FLAG-MAVS and pcD-NSP1. NSP1 was found to co-immunoprecipitated with MAVS both during viral infection and overexpression suggesting an interaction in absence of other viral protein. Deletion mutant of NSP1 suggested that the C-terminal of NSP1 was sufficient for its interaction with MAVS; however as the RING domain is in N-terminal, a full length NSP1 was necessary for MAVS degradation. Previous studies by Qin *et al* had also reported through luciferase assay that NSP1ΔIRF3 binding domain is incapable of downregulating MAVS induced IFN-β. This might be explained, since removing the IRF3-BD domain of NSP1 hampers its MAVS downregulating property. Furthermore mutants of MAVS were constructed to identify the domain responsible for binding with NSP1. MAVS protein comprises of a typical caspase activation and recruitment domain (CARD) at its N-terminal followed by a proline-rich region (Pro) in the middle and a hydrophobic transmembrane (TM) domain at the C terminus. Co-IP studies revealed that CARD-TM domain was necessary for binding with NSP1 whereas Pro region did not interact with NSP1 (Figure 4D). In addition to binding with NSP1, CARD-TM domain alone could inhibit IFN-β transcription in presence of NSP1 similar to full length MAVS.

Recently it was shown by Hou *et al* [5] that in response to viral infection, RIG-I-like RNA helicases bind to viral RNA and activate the mitochondrial protein MAVS. This activation leads to the formation of very large MAVS aggregates on the mitochondrial membrane which interacts with cytosolic signaling proteins, such as TRAFs, resulting in the activation of IKKs and TBK1. These MAVS aggregates were detergent resistant in nature and therefore a method called semidenaturing detergent agarose gel electrophoresis (SDD-AGE) was employed, which was previously used for the detection of prion-like structures. Since A5-16 (NSP1 mutant), strain did not degrade MAVS; MAVS aggregates were studied followed A5-16 infection. A smear of SDS- resistant high molecular weight MAVS was observed in time dependent manner during RV infection (Figure 8A). No change in overall MAVS expression was observed by immunoblotting. Similar results were obtained when HEK293 cells were transiently transfected with FLAG-MAVS and infected with A5-16. MAVS aggregates were abrogated in presence of NSP1 but were restored when MG132 was added. Restoration of MAVS aggregates in presence of MG132 further confirms that MAVS degradation by NSP1 is proteasome mediated.

In most mammalian cells, activation of IRF3 leads to the expression of IFN-β, which in turn induces the expression of master regulator IRF7. By targeting IRF3 for degradation, NSP1 removes an upstream transcription factor needed for IRF7 expression, thereby subverting the production of type I IFN. The dual mechanism of NSP1 in subverting IRF7 function may be crucial for successful virus replication in the host at later stages of infection, where the virus may be challenged in cells that have transitioned from a naive to an antiviral status due to exposure to cytokines or debris from neighboring infected cells. However, by degrading MAVS, RV may escape the initial antiviral defense mechanisms activated by the RIGI–MDA5 pathway. During RV infection there is a potential redundancy in the functions of RIG-I and MDA-5 [18], therefore abrogating MAVS results in a complete shutdown of the host RNA sensory machinery.

**Figure 9:**
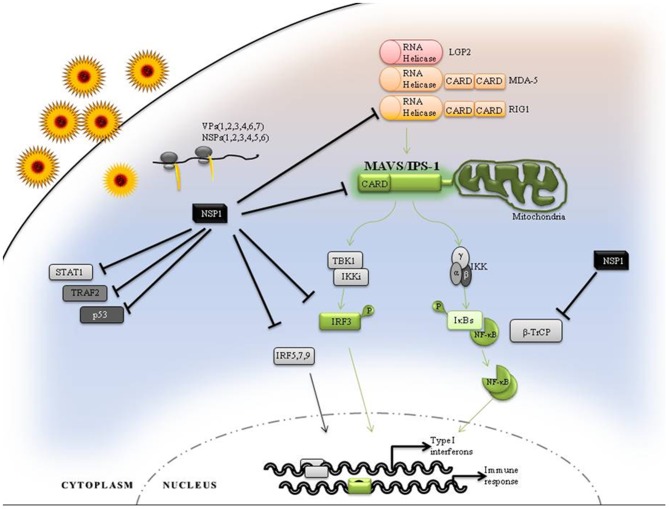
Mechanistic model for rotavirus NSP1-mediated attenuation of cellular proteins for improved infection. The model summarizes all the NSP1 mediated inhibitory effects during RV infection. In brief, it shows the critical role of MAVS in RLR mediated activation of type I IFN and immune response. By degrading MAVS, NSP1 can directly inhibit IRF3 and NF-κB signaling. Other cellular proteins like IRFs and β-TrCP are targeted selectively but MAVS degradation was observed in all RV strains with functional NSP1.

Based on the results obtained in this study as well as previous reports, we propose a model for the NSP1 mediated modulation of RV innate immune response (Figure 9). Briefly, RV cell entry and subsequent replication leads to MDA-5 and RIG-I activation which may be activated by different types of viral PAMPs [18]. Initially the adaptor MAVS associates with activated PRRs and propagates signaling to activate IRF3 and NF-κB. But during RV infection, expression of NSP1 leads to the degradation of RIGI, MAVS, IRF3 and IRF7 and thus IFN-β secretion. For most of the animal RV strains where NSP1 effectively degrades IRF3, the primary antiviral response is inhibited significantly which is further accentuated by degradation of MAVS protein. In case of RV strains, such as OSU, Wa, DS-1 or KU which cannot efficiently degrade IRF3, down regulation of MAVS results in a sustained inhibition of IFN-β secretion to maintain pro-viral state in cell. Overall, all targets of NSP1 directly or individually inhibit IFN activation, suggesting multiple strategies to evade IRF-dependent and -independent signaling pathways. Further understanding of these mechanisms should yield novel strategies for developing antivirals that evoke responses to eliminate RV infection.

## Supporting Information

Figure S1 **The inhibition of viral replication induced by UV treatment.** To prepare UV-inactivated RV, simian SA11 were pretreated with 40 μg/ml psoralen AMT and then irradiated by long-wave UV-light (365 nm) for 2 hours. HT29 cells were infected with SA11 or UV-SA11at 1 M.O.I. for indicated time points. **(A)** RNA was isolated at specific intervals followed by quantification of *nsp4* and *gapdh* mRNA transcripts by qRT-PCR. Fold changes were obtained by normalizing relative gene expressions to gapdh using the formula 2^−ΔΔCT^ (ΔΔCT = ΔC_TSample_-ΔC_TUntreated control_). **(B)** At indicated time points, HT29 cells infected with normal and UV irradiated virus were freeze-thawed. Extracted and purified viral preparations were titrated by plaque assay.
(TIF)

Figure S2 **Effect of MAVS degradation induced by NSP1 on IRF3-5D overexpression.** With an aim to analyze the effect of NSP1 on MAVS during constitutively phosphorylated IRF3, vector encoding IRF3-5D (a phosphomimetic form of IRF3) was overexpressed, along with NSP1 in presence and absence of MAVS followed by immunoblotting with p-IRF3 antibody In presence of both NSP1 and MAVS, the p-IRF3 levels induced by IRF3-5D overexpression, were significantly inhibited compared to only MAVS and IRF3-5D transfected cells. Membranes were reprobed with IRF3 and GAPDH antibodies.
(TIF)

Figure S3 **NSP1 mediated degradation of Mini-MAVS (pFLAG-CARD-TM).** In order to co-relate the interaction between CARD-TM and NSP1, HEK293 cells were transfected with vectors encoding mini-MAVS and increasing concentration of pcD-NSP1 for 24 hours. Results reveal dose-dependent degradation of CARD-TM. Membranes were probed with anti-FLAG, anti-His and to confirm equal loading GAPDH antibodies.
(TIF)
