# Correction: MAVS Protein Is Attenuated by Rotavirus Nonstructural Protein 1

**DOI:** 10.1371/journal.pone.0131956

**Published:** 2015-06-25

**Authors:** Satabdi Nandi, Shampa Chanda, Parikshit Bagchi, Mukti Kant Nayak, Rahul Bhowmick, Mamta Chawla-Sarkar, The PLOS ONE Staff

After the publication of the article by Nandi et al. [1] a number of concerns were raised about the figures and aspects of the reporting in the article. After follow up on the concerns raised, the PLOS staff editors and the authors issue this Correction, and the authors apologize for the errors in the published article. The authors have provided detailed explanations of the discrepancies and the raw blots, and they have conducted a replication study. The first author takes sole responsibility for the errors in figure handling, preparation, and labelling and apologizes to the editors and readers.

The concerns noted about the date reported are outlined below and have also been raised to the attention of the authors' institution, the National Institute of Cholera and Enteric Diseases:

1. Lane duplication in Figures 1C and 2B: The authors have acknowledged that the GAPDH lanes were duplicated in error by the first author. The authors sincerely apologize for this error.
2. Lane duplication in Figures 5A and 5C: The authors have acknowledged that the GAPDH lanes were duplicated in error by the first author. The authors sincerely apologize for this error.
3. Virus inactivation: the authors used UV-inactivated SA11 rotavirus in the experiments reported in Fig 1C. According to Groene WS, Shaw RD: Psoralen preparation of antigenically intact noninfectious rotavirus particles. J Virol Methods. 1992 Jul;38(1):93–102, this virus should not be able to replicate (and this is shown in S1 Fig). The present study does not explain the presence of non-structural proteins NSP1 and NSP3.
  The author’s response is as follows: UV inactivation of SA11 does not totally inactivate the virus; around 85–95% inactivation is obtained, depending on the experiment. Therefore, we can see very faint bands for NSP1 and NSP3. In a lighter exposure, one would not see any NSP1 or NSP3 bands. The blot is shown as it appeared in the first exposure.
4. Virus titer: S1B Fig shows that the infectious SA11 virus titer was only 1.5 log at 20h after infection at a multiplicity of infection of 1, i.e. <100 plaque-forming units/ml. The titer would be expected to be several orders of magnitude higher.
  The author’s response is as follows: S1B Fig was not labeled correctly as the axis was meant to be PFU/ml X 10^6 (1.5 X 10^6). In HT29 cells, SA11 replicates much more slowly compared to in MA104 cells, in which the authors have also seen and reported higher PFU values at 20h. A corrected figure has been provided.

At the journal’s request the authors provided copies of the original uncropped and unadjusted blots for all of the Western blots. Most of the blots represent the same image as the published article, but in some cases alternate blots from replicate experiments or different exposures of the same experiment have been provided. The authors state that in experiments where ChemiDoc was used instead of the usual film development, some images have been cropped and the original exposure not saved.

Upon evaluation of the raw blots provided by the authors, while the same overall trends in results were observed, the editors had concerns that they did not fully match the following published figures: 2A, 2C, MAVS and GAPDH panels in Figure 2D, 3A, 5A, 5C, right panel in Figure 6A, 7, S3.

The authors have conducted an internal replication study. Several experiments were repeated in conditions kept as similar as possible to those in the published study, though the virus stock and cell line passage numbers were different. The strains and time points used were same. The editors believe that these results from the published article are reproduced by this internal replication study. The blots replicated the following figures from the article:

Fig 1C, lanes MAVS, NSP3, GAPDH

Fig 1E, all lanes

Fig 2A, lanes MAVS, NSP3, GAPDH

Fig 2B, lanes MAVS, NSP1, NSP3, GAPDH

Fig 2C, lanes MAVS, NSP3, GAPDH

Fig 3A, all lanes

Fig 5C, lanes P-S396-IRF3, GAPDH

Fig 6A, left panel, all lanes

Fig 6B, left panel, all lanes

Fig 8A, all lanes

In the article, "psoralane" should be "psoralen".

## Supporting Information

S1 Fig The inhibition of viral replication induced by UV treatment. To prepare UV-inactivated RV, simian SA11 were pretreated with 40 μg/ml psoralen AMT and then irradiated by long-wave UV-light (365 nm) for 2 hours. HT29 cells were infected with SA11 or UV-SA11at 1 M.O.I. for indicated time points. (A) RNA was isolated at specific intervals followed by quantification of *nsp4* and *gapdh* mRNA transcripts by qRT-PCR. Fold changes were obtained by normalizing relative gene expressions to gapdh using the formula 2^−ΔΔCT^(ΔΔCT =  ΔC_TSample_-ΔC_TUntreated control_). (B) At indicated time points, HT29 cells infected with normal and UV irradiated virus were freeze-thawed. Extracted and purified viral preparations were titrated by plaque assay.
(TIF)

S1 File Raw Blots. (ZIP)

S2 File Replication Images. (ZIP)
