# Supplementary figures and images for: MAVS Protein Is Attenuated by Rotavirus Nonstructural Protein 1
Source: PLoS One. 2014 Mar 18;9(3):e92126. doi: 10.1371/journal.pone.0092126 (PMC3958477; doi:10.1371/journal.pone.0092126)

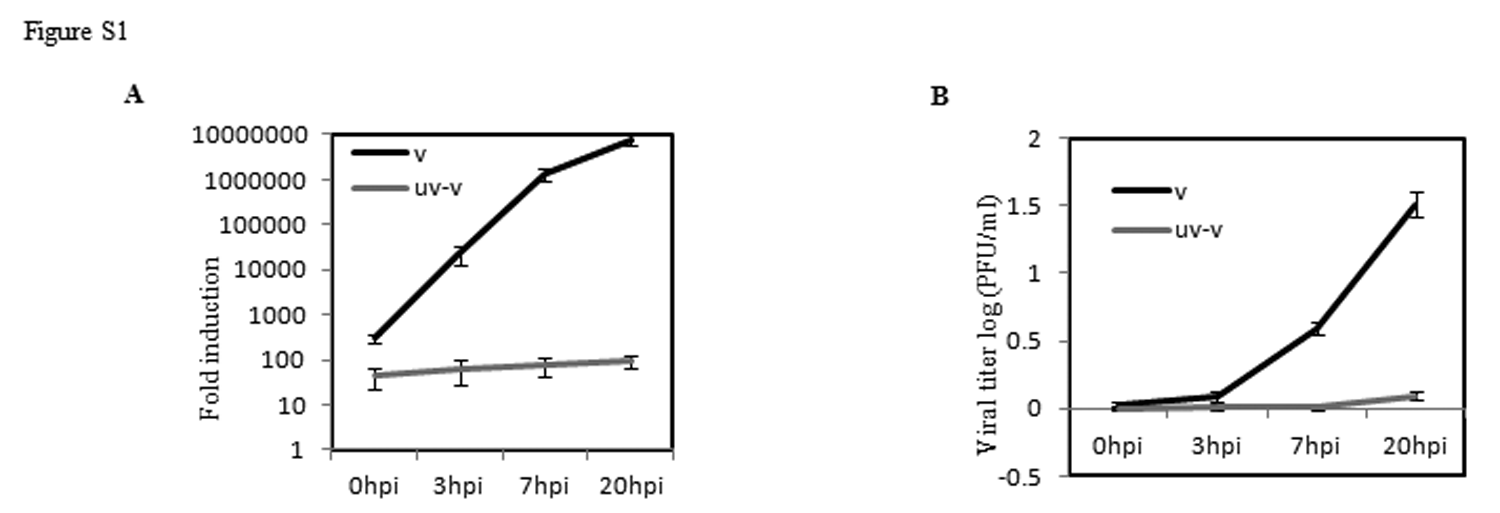

Supplement: Figure S1 — The inhibition of viral replication induced by UV treatment. To prepare UV-inactivated RV, simian SA11 were pretreated with 40 μg/ml psoralen AMT and then irradiated by long-wave UV-light (365 nm) for 2 hours. HT29 cells were infected with SA11 or UV-SA11at 1 M.O.I. for indicated time points. (A) RNA was isolated at specific intervals followed by quantification of nsp4 and gapdh mRNA transcripts by qRT-PCR. Fold changes were obtained by normalizing relative gene expressions to gapdh using the formula 2−ΔΔCT (ΔΔCT = ΔCTSample-ΔCTUntreated control). (B) At indicated time points, HT29 cells infected with normal and UV irradiated virus were freeze-thawed. Extracted and purified viral preparations were titrated by plaque assay. (TIF) [file pone.0092126.s001.tif]

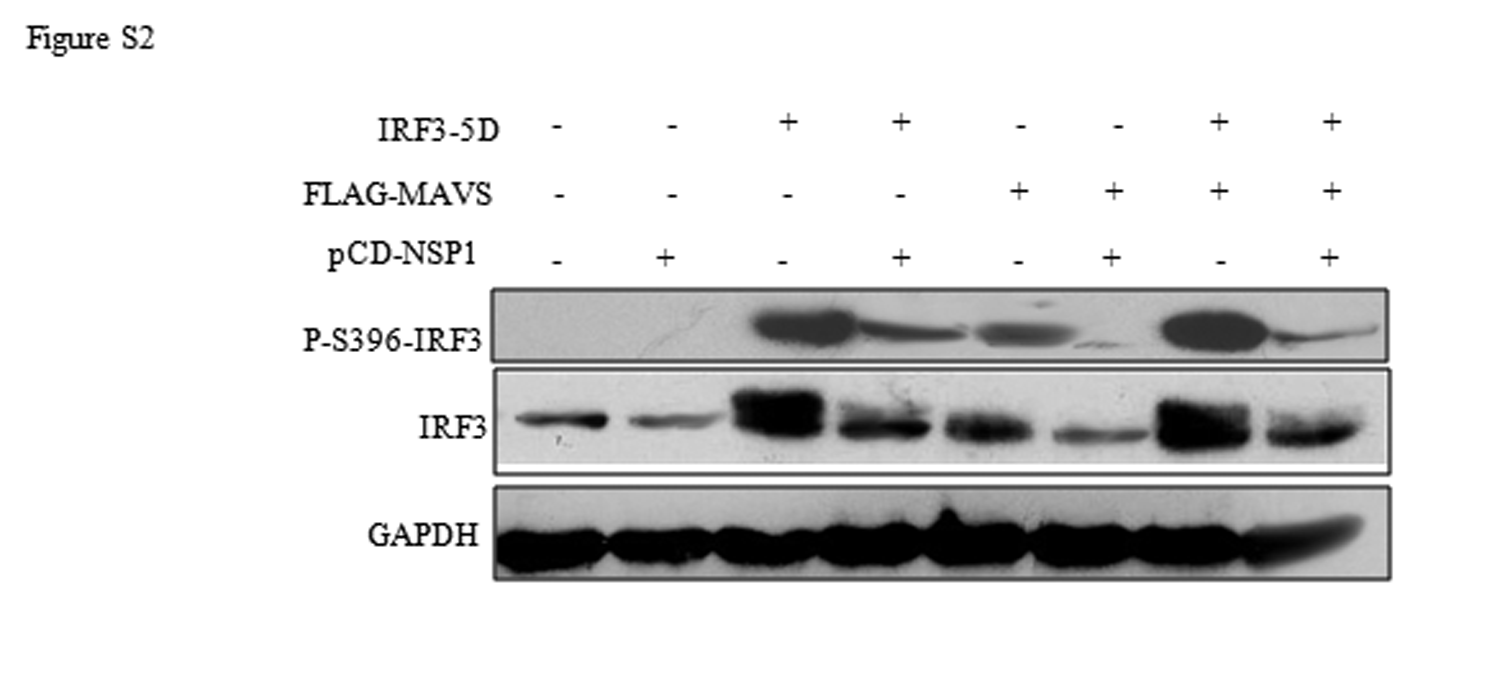

Supplement: Figure S2 — Effect of MAVS degradation induced by NSP1 on IRF3-5D overexpression. With an aim to analyze the effect of NSP1 on MAVS during constitutively phosphorylated IRF3, vector encoding IRF3-5D (a phosphomimetic form of IRF3) was overexpressed, along with NSP1 in presence and absence of MAVS followed by immunoblotting with p-IRF3 antibody In presence of both NSP1 and MAVS, the p-IRF3 levels induced by IRF3-5D overexpression, were significantly inhibited compared to only MAVS and IRF3-5D transfected cells. Membranes were reprobed with IRF3 and GAPDH antibodies. (TIF) [file pone.0092126.s002.tif]

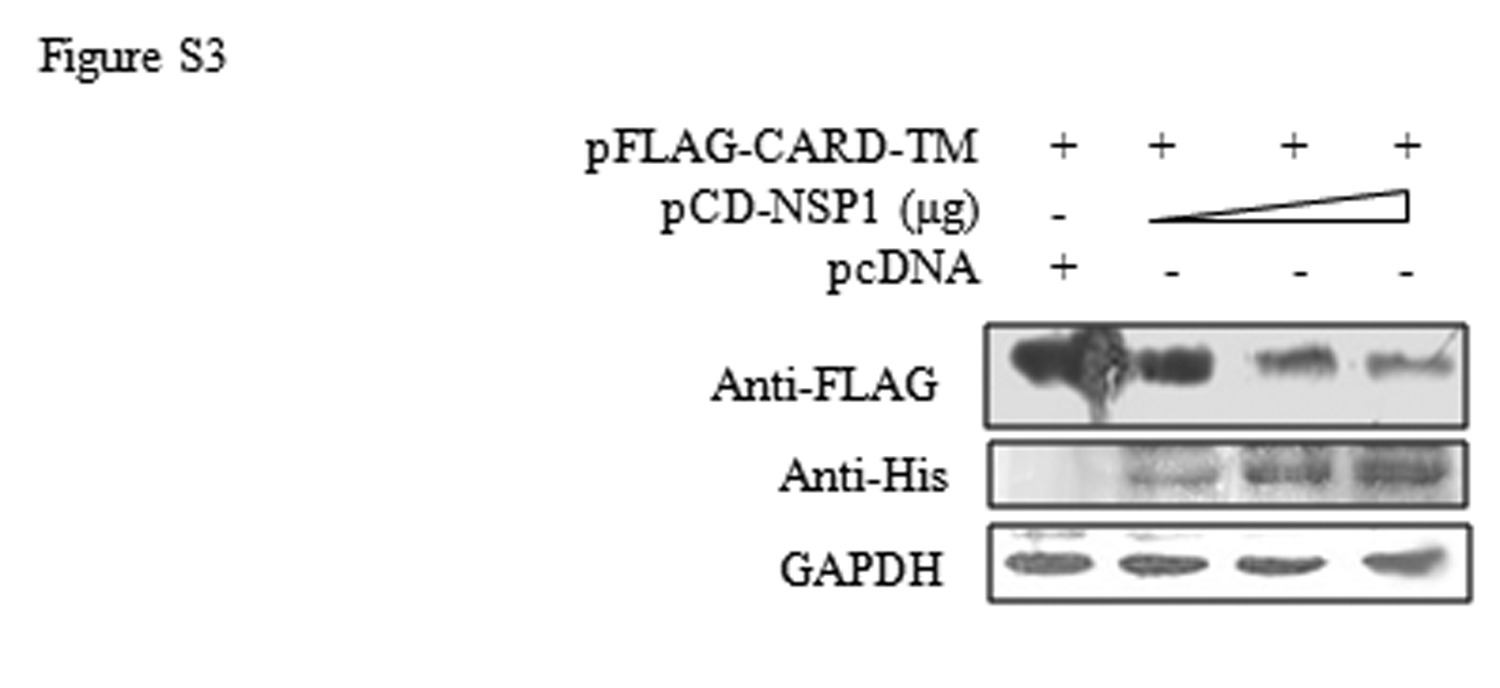

Supplement: Figure S3 — NSP1 mediated degradation of Mini-MAVS (pFLAG-CARD-TM). In order to co-relate the interaction between CARD-TM and NSP1, HEK293 cells were transfected with vectors encoding mini-MAVS and increasing concentration of pcD-NSP1 for 24 hours. Results reveal dose-dependent degradation of CARD-TM. Membranes were probed with anti-FLAG, anti-His and to confirm equal loading GAPDH antibodies. (TIF) [file pone.0092126.s003.tif]
