# Supplementary figures and images for: Correction: MAVS Protein Is Attenuated by Rotavirus Nonstructural Protein 1
Source: PLoS One. 2015 Jun 25;10(6):e0131956. doi: 10.1371/journal.pone.0131956 (PMC4482503; doi:10.1371/journal.pone.0131956)

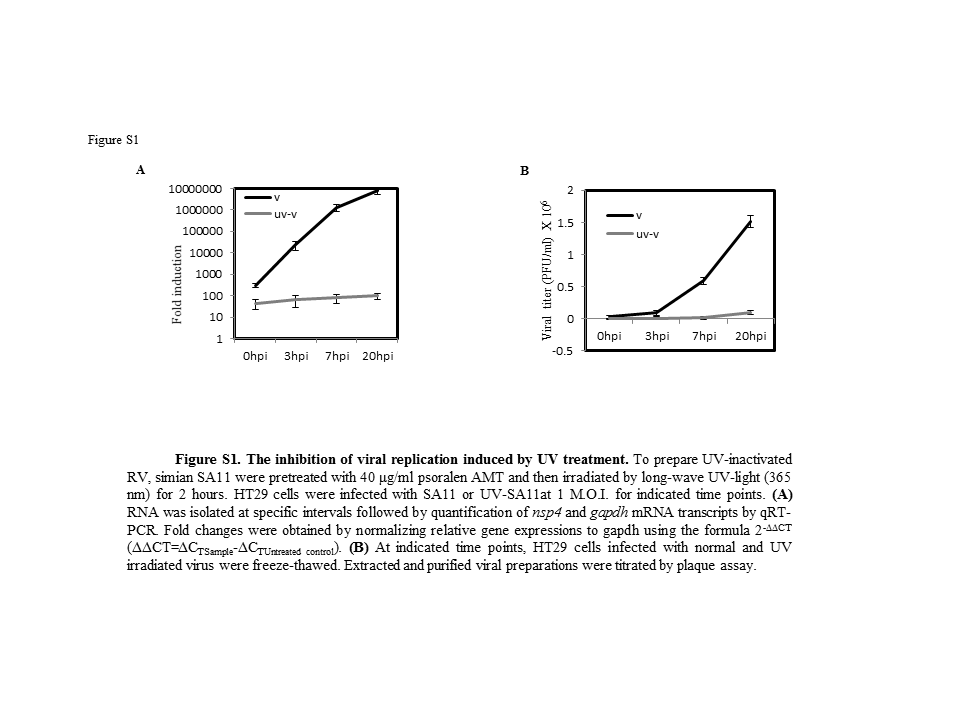

Supplement: S1 Fig — To prepare UV-inactivated RV, simian SA11 were pretreated with 40 μg/ml psoralen AMT and then irradiated by long-wave UV-light (365 nm) for 2 hours. HT29 cells were infected with SA11 or UV-SA11at 1 M.O.I. for indicated time points. (A) RNA was isolated at specific intervals followed by quantification of nsp4 and gapdh mRNA transcripts by qRT-PCR. Fold changes were obtained by normalizing relative gene expressions to gapdh using the formula 2−ΔΔCT(ΔΔCT = ΔCTSample-ΔCTUntreated control). (B) At indicated time points, HT29 cells infected with normal and UV irradiated virus were freeze-thawed. Extracted and purified viral preparations were titrated by plaque assay. (TIF) [file pone.0131956.s001.TIF]

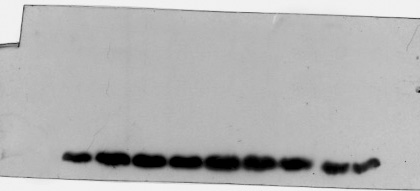

Supplement: S1 File — (ZIP) [file pone.0131956.s002.zip › Gapdh-Fig2B (raw).jpg]

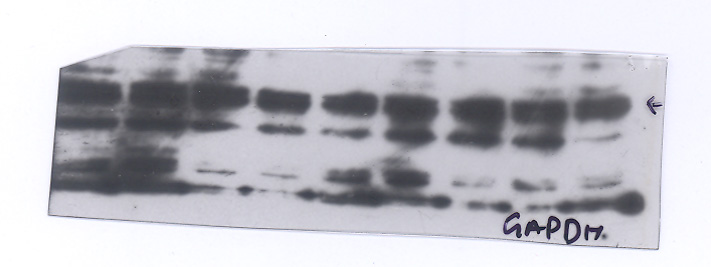

Supplement: S1 File — (ZIP) [file pone.0131956.s002.zip › GAPDH Fig-5C [raw] (used Blot).jpg]

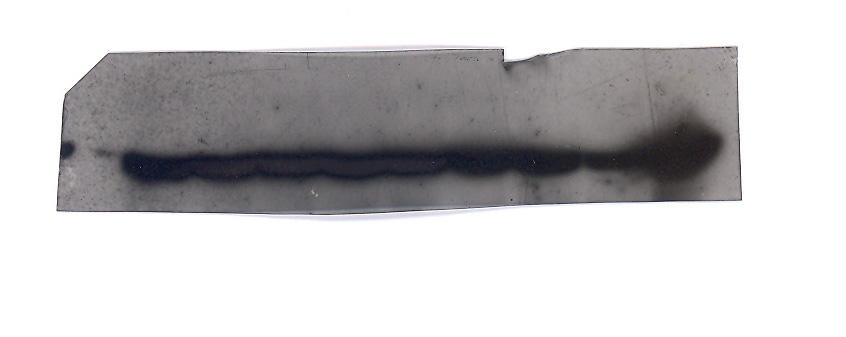

Supplement: S1 File — (ZIP) [file pone.0131956.s002.zip › Gapdh Fig-1C [raw] (used Blot).jpg]

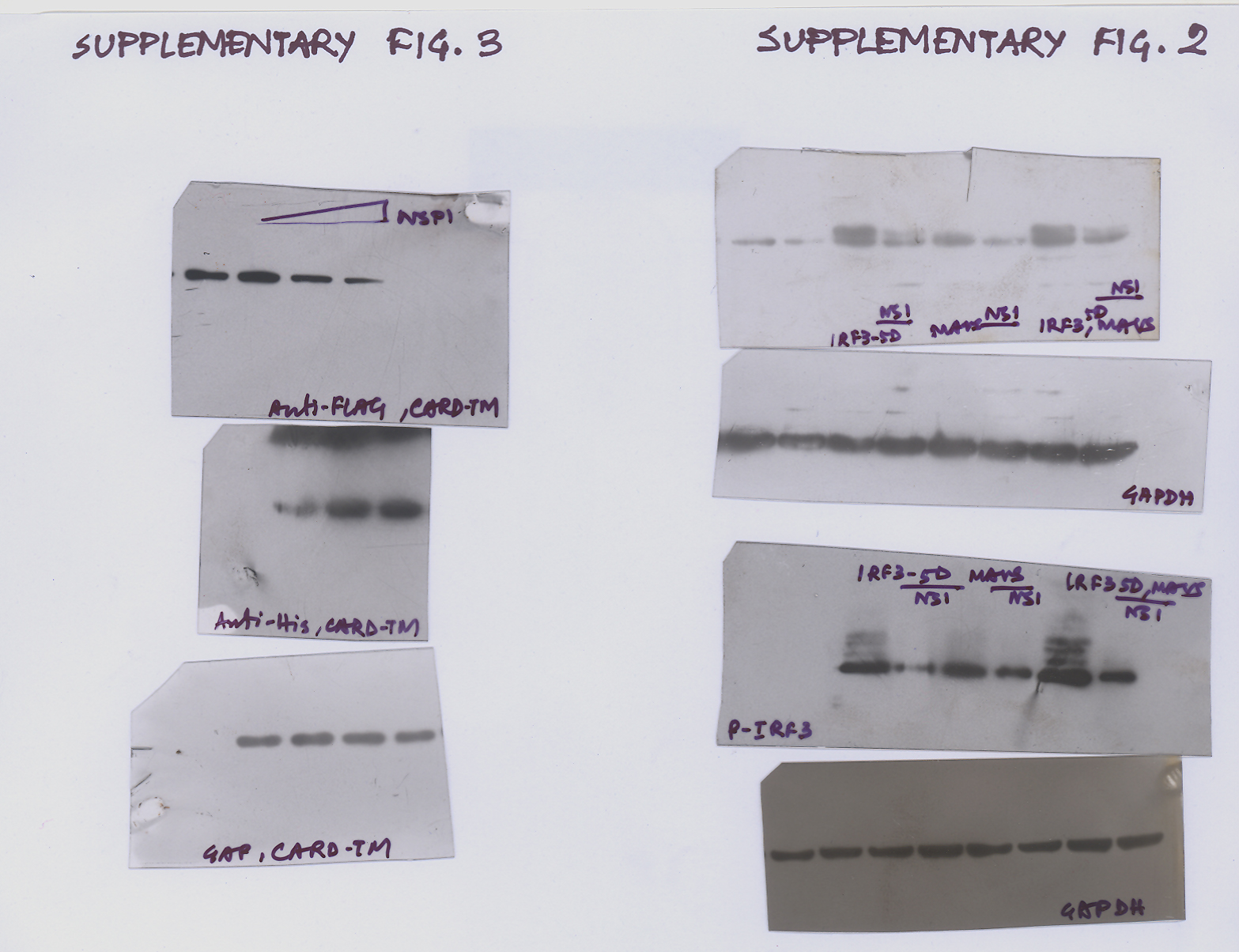

Supplement: S1 File — (ZIP) [file pone.0131956.s002.zip › SUPPLEMENTARY FIGURE-2,3.jpg]

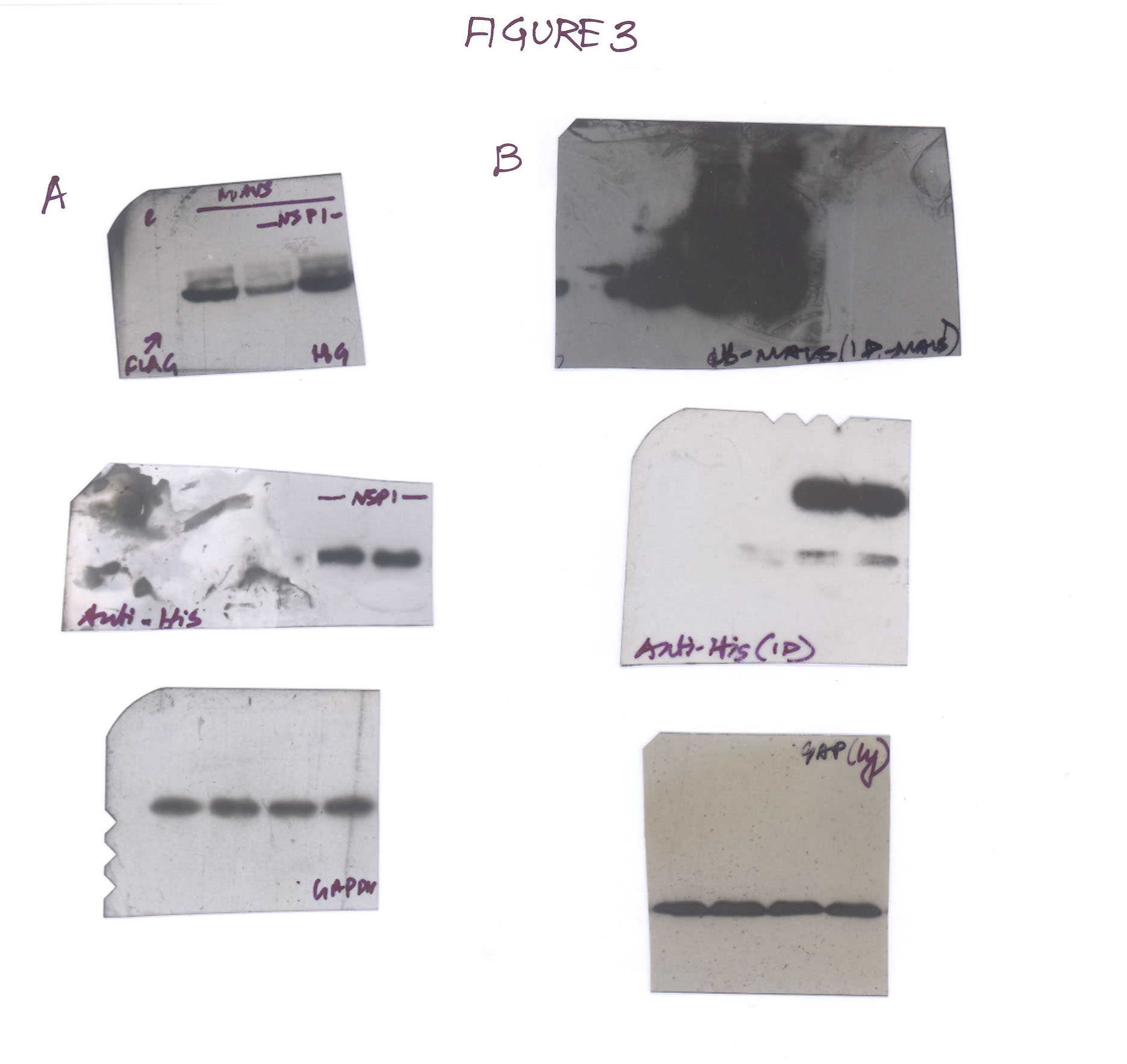

Supplement: S1 File — (ZIP) [file pone.0131956.s002.zip › FIGURE 3.jpg]

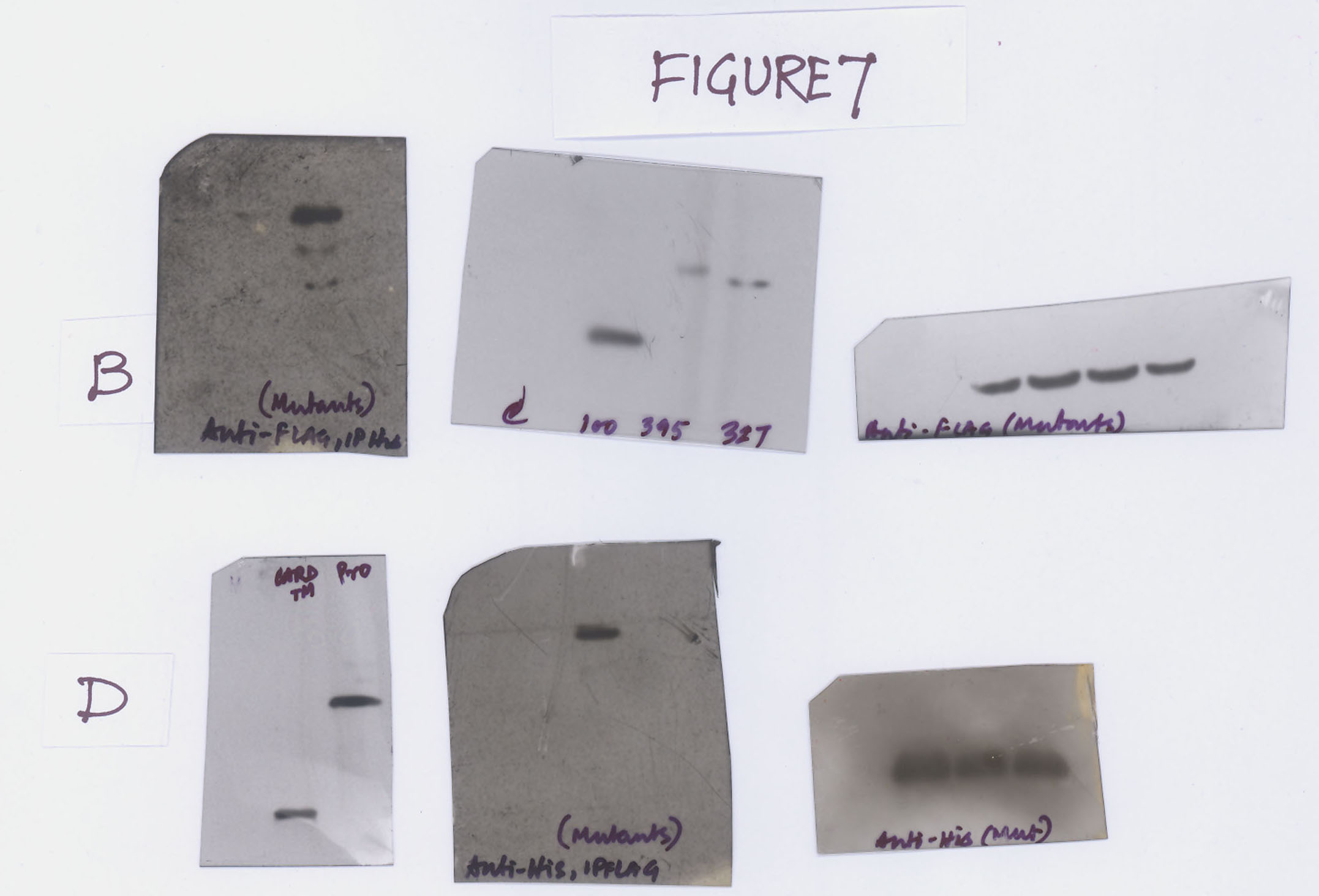

Supplement: S1 File — (ZIP) [file pone.0131956.s002.zip › FIGURE 7.jpg]

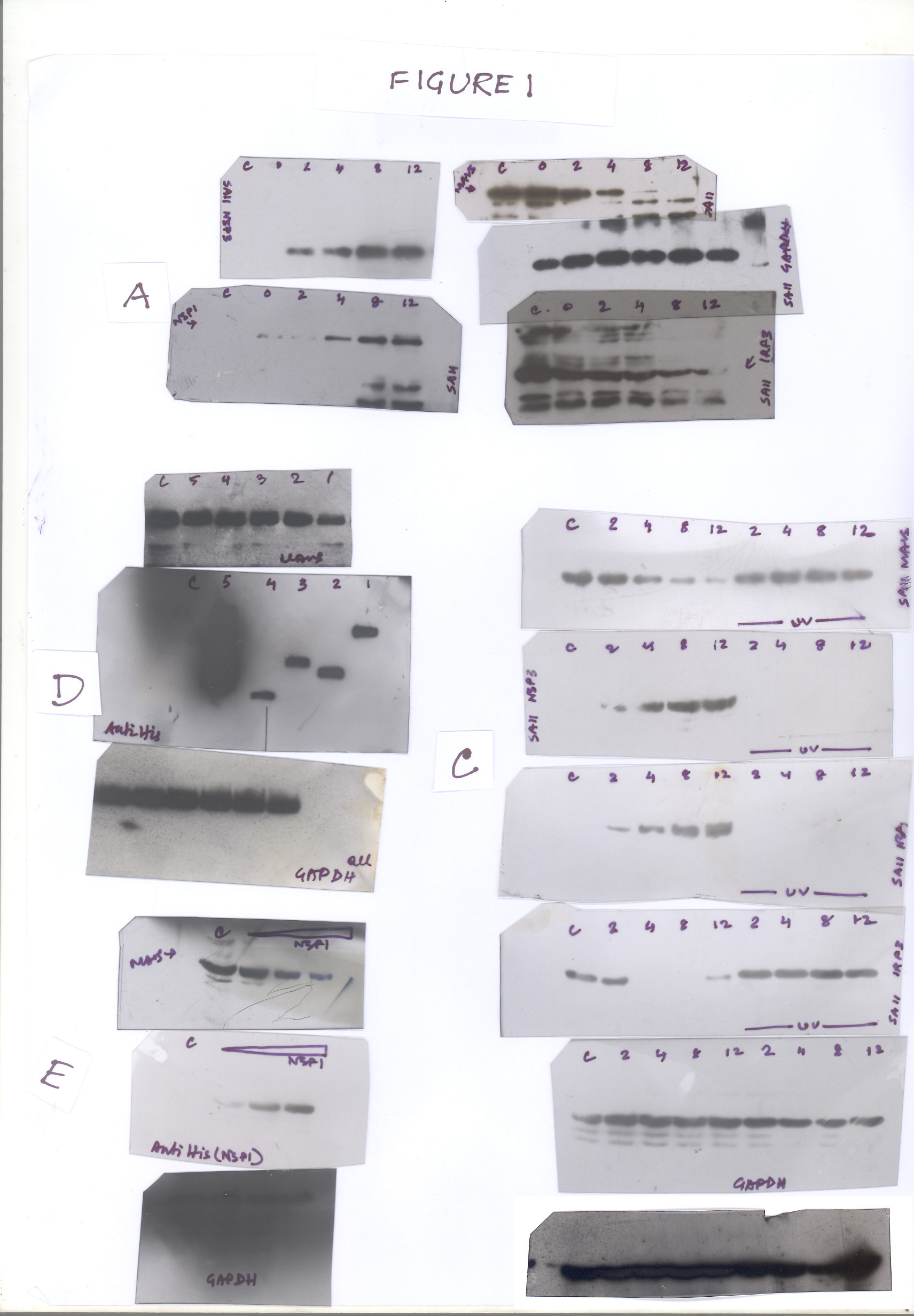

Supplement: S1 File — (ZIP) [file pone.0131956.s002.zip › FIGURE-1.jpg]

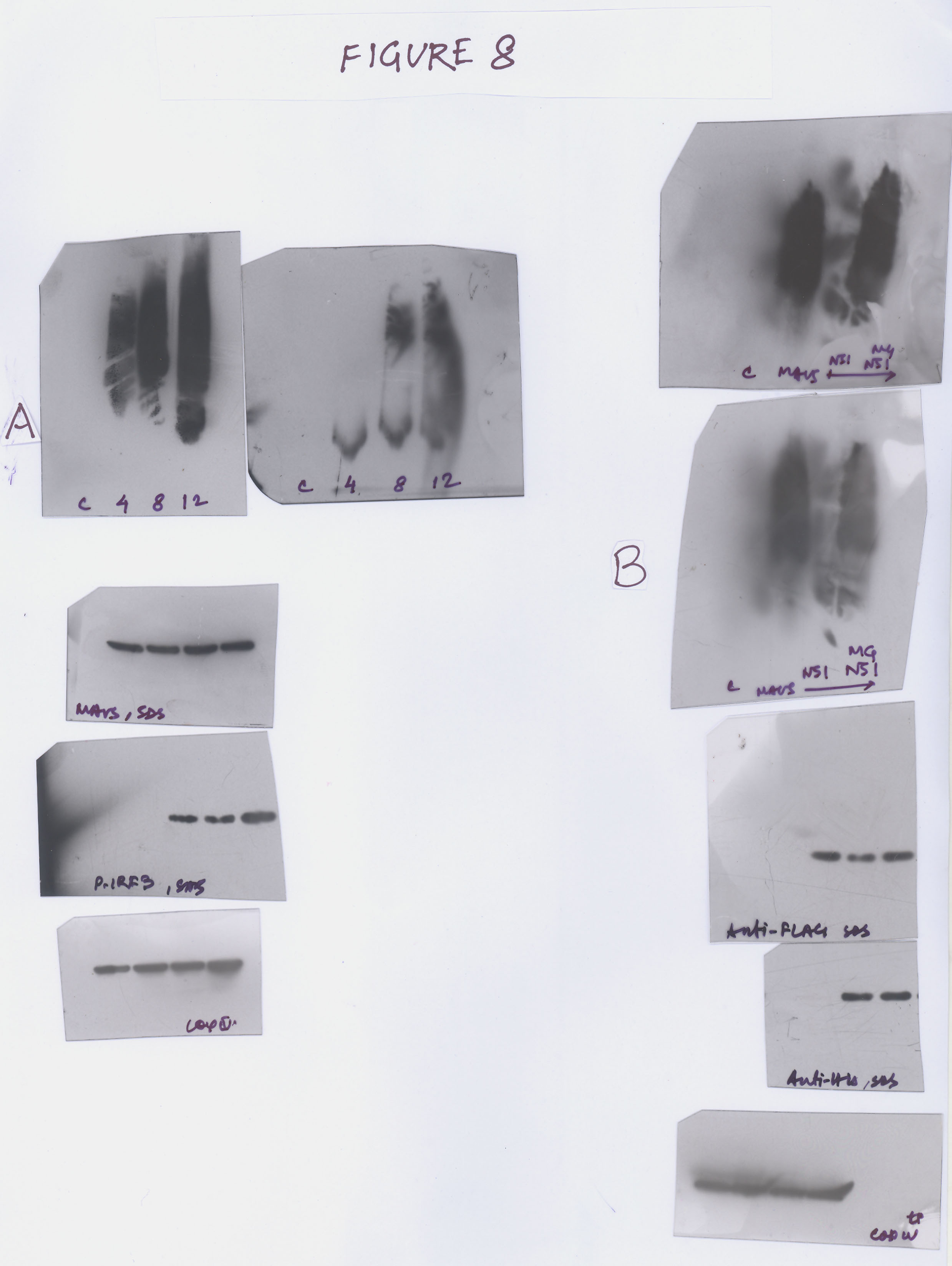

Supplement: S1 File — (ZIP) [file pone.0131956.s002.zip › FIGURE-8.jpg]

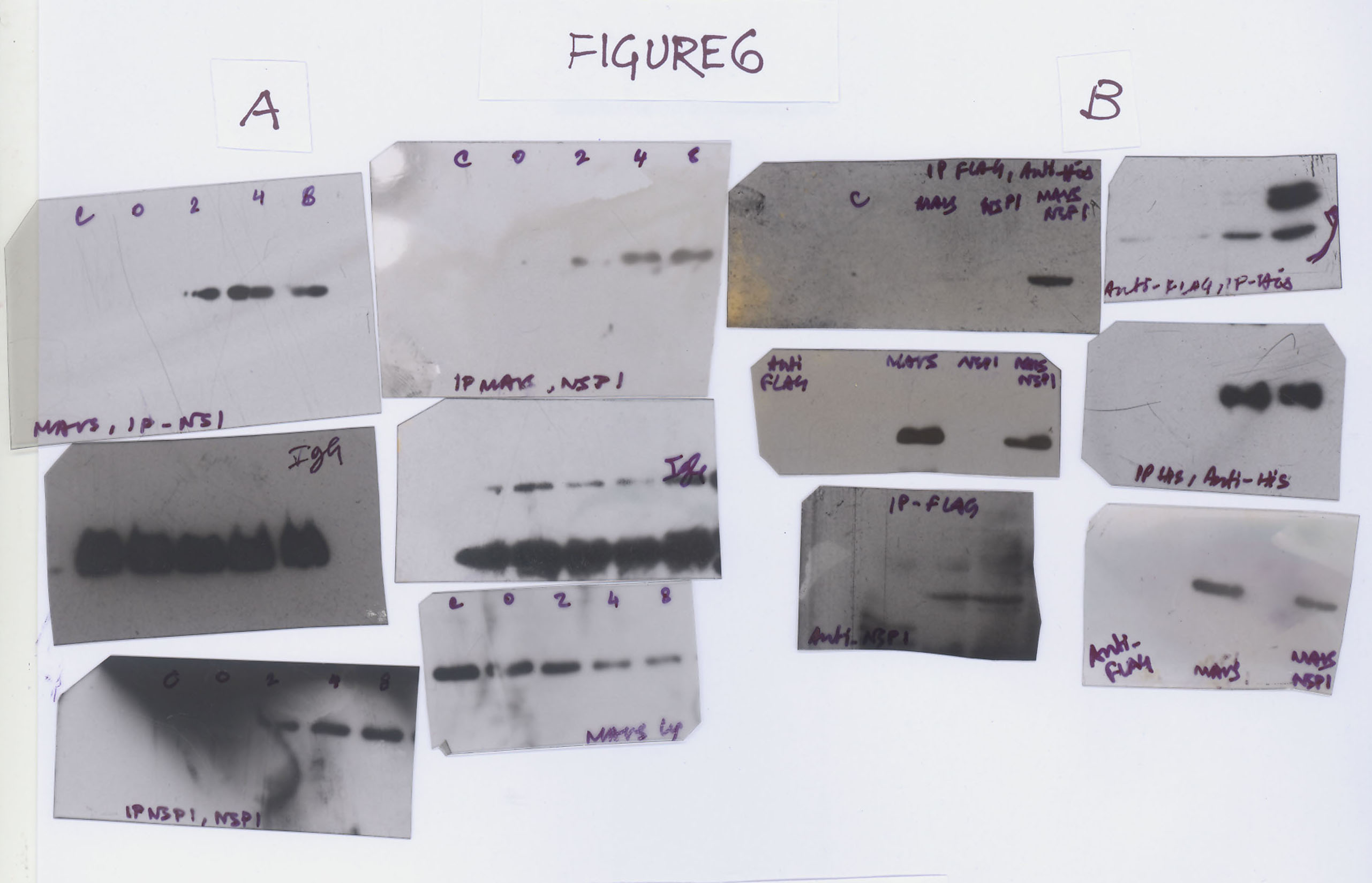

Supplement: S1 File — (ZIP) [file pone.0131956.s002.zip › FIGURE 6.jpg]

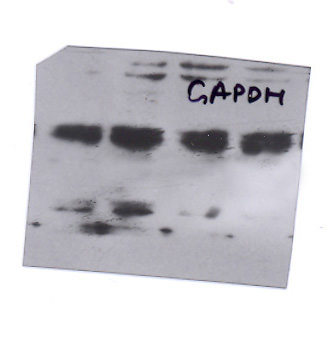

Supplement: S1 File — (ZIP) [file pone.0131956.s002.zip › GAPDH Fig-5A [raw] (Used blot).jpg]

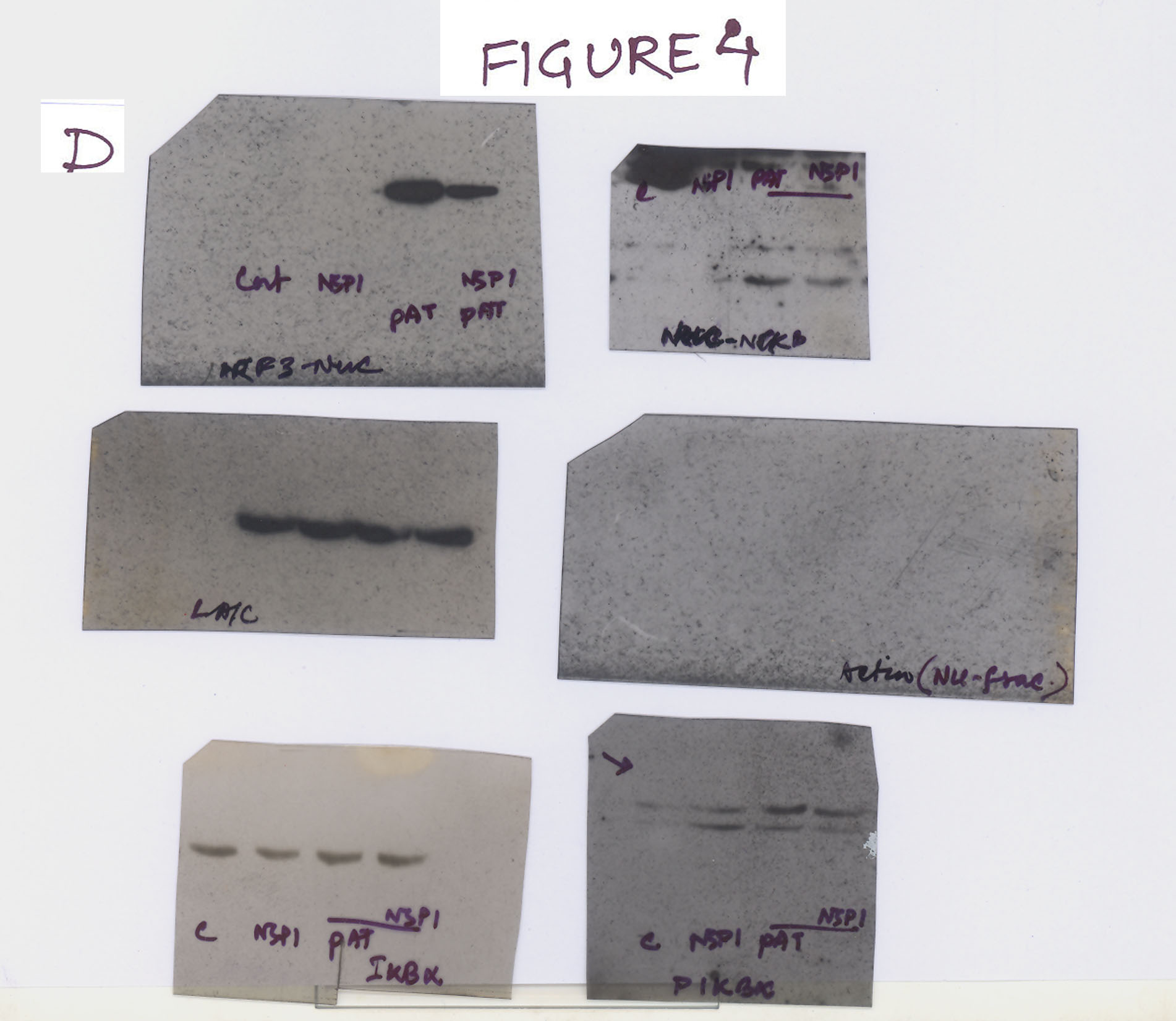

Supplement: S1 File — (ZIP) [file pone.0131956.s002.zip › FIGURE 4.jpg]

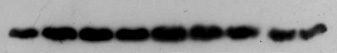

Supplement: S1 File — (ZIP) [file pone.0131956.s002.zip › Gapdh-Fig2B (crop).jpg]

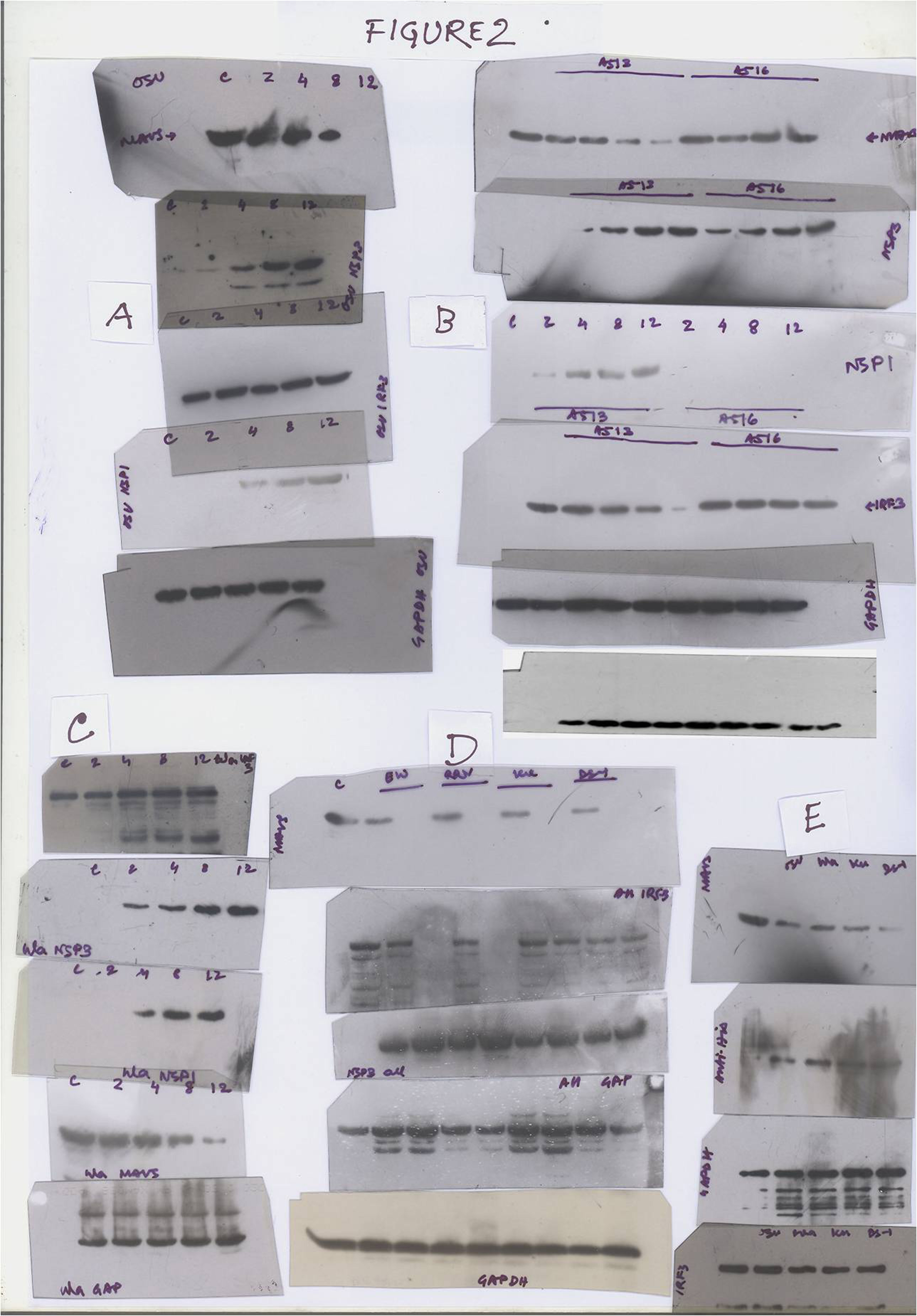

Supplement: S1 File — (ZIP) [file pone.0131956.s002.zip › FIGURE-2.jpg]

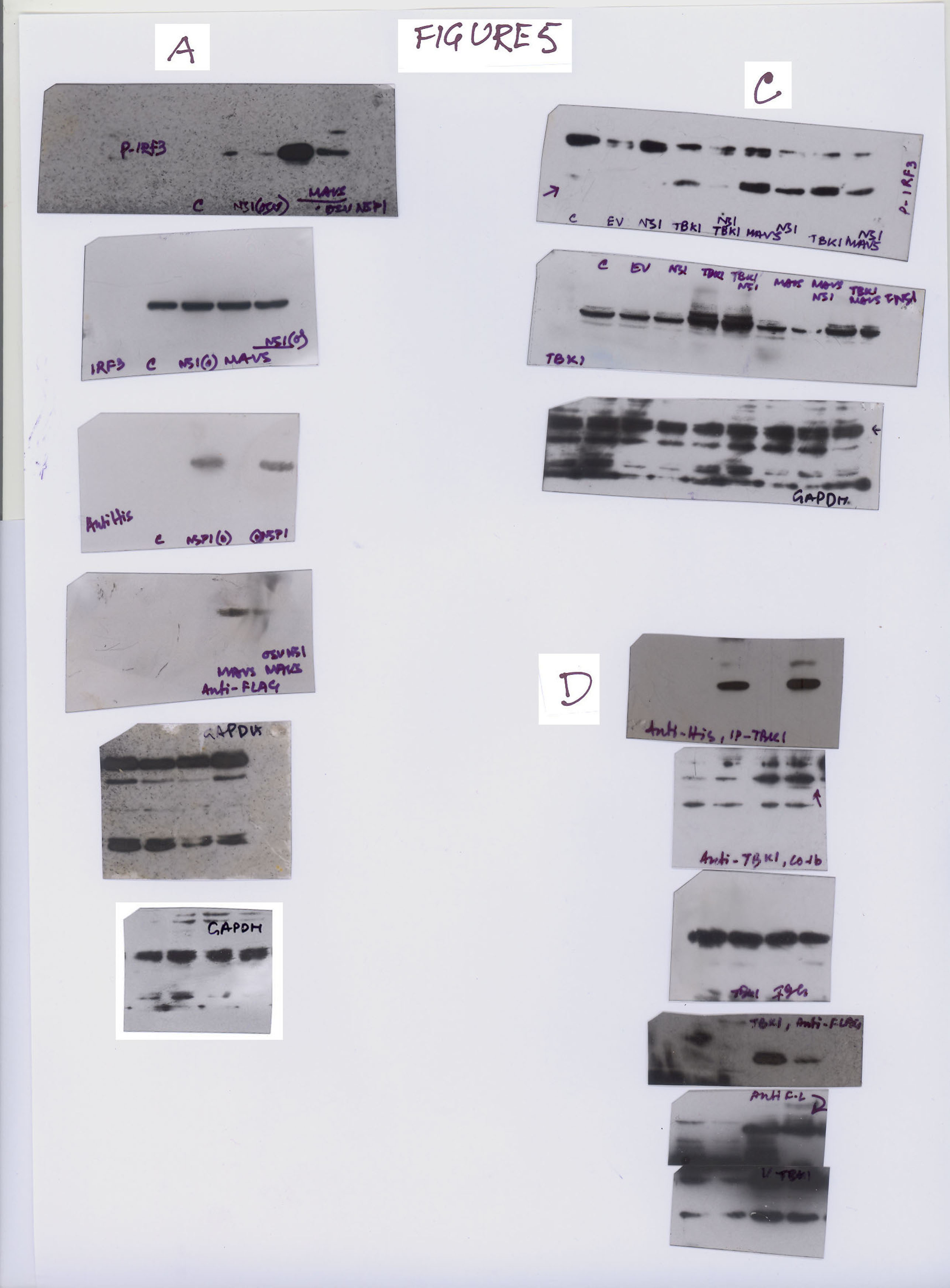

Supplement: S1 File — (ZIP) [file pone.0131956.s002.zip › FIGURE 5.jpg]

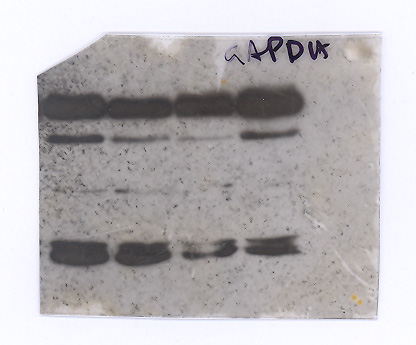

Supplement: S1 File — (ZIP) [file pone.0131956.s002.zip › GAPDH Fig-5A (duplicate experiment).jpg]

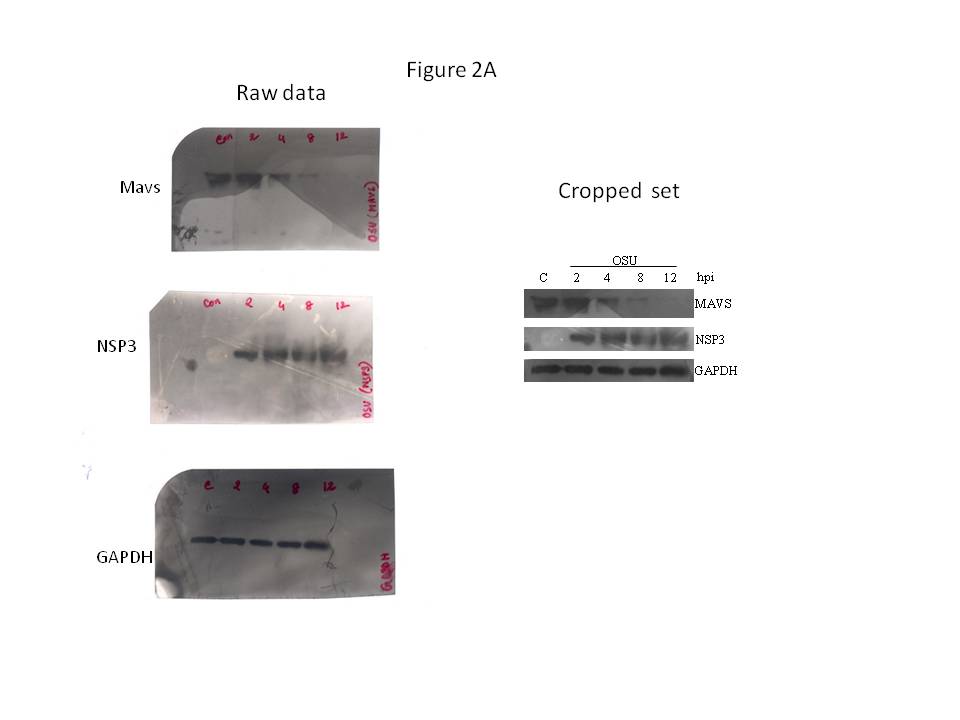

Supplement: S2 File — (ZIP) [file pone.0131956.s003.zip › Fig 2A.JPG]

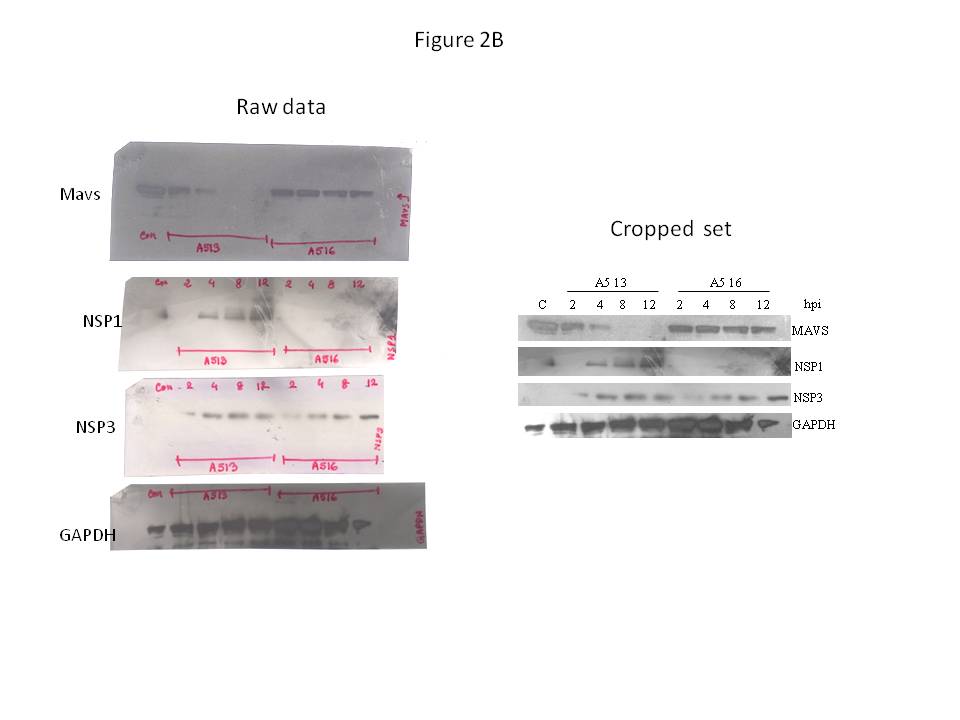

Supplement: S2 File — (ZIP) [file pone.0131956.s003.zip › Fig 2B.JPG]

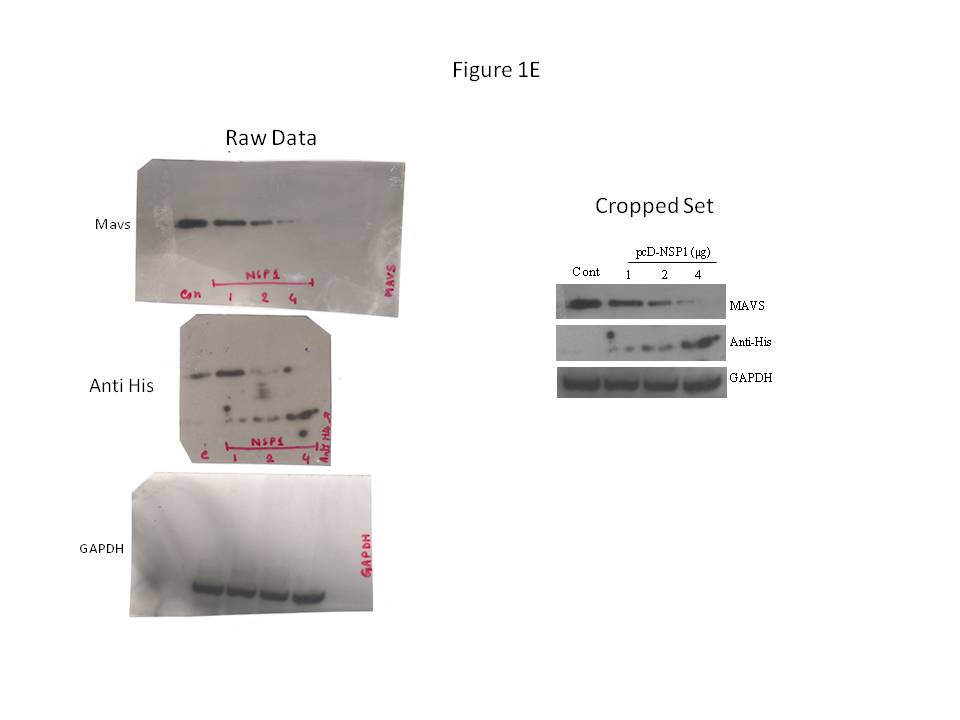

Supplement: S2 File — (ZIP) [file pone.0131956.s003.zip › Fig 1E.JPG]

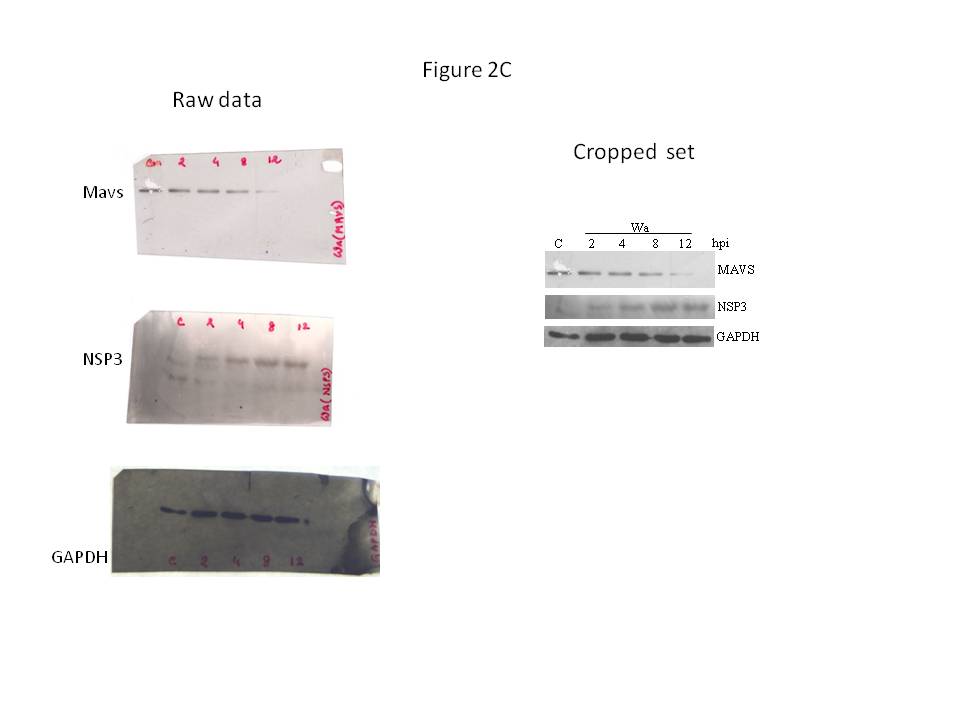

Supplement: S2 File — (ZIP) [file pone.0131956.s003.zip › Fig 2C.JPG]

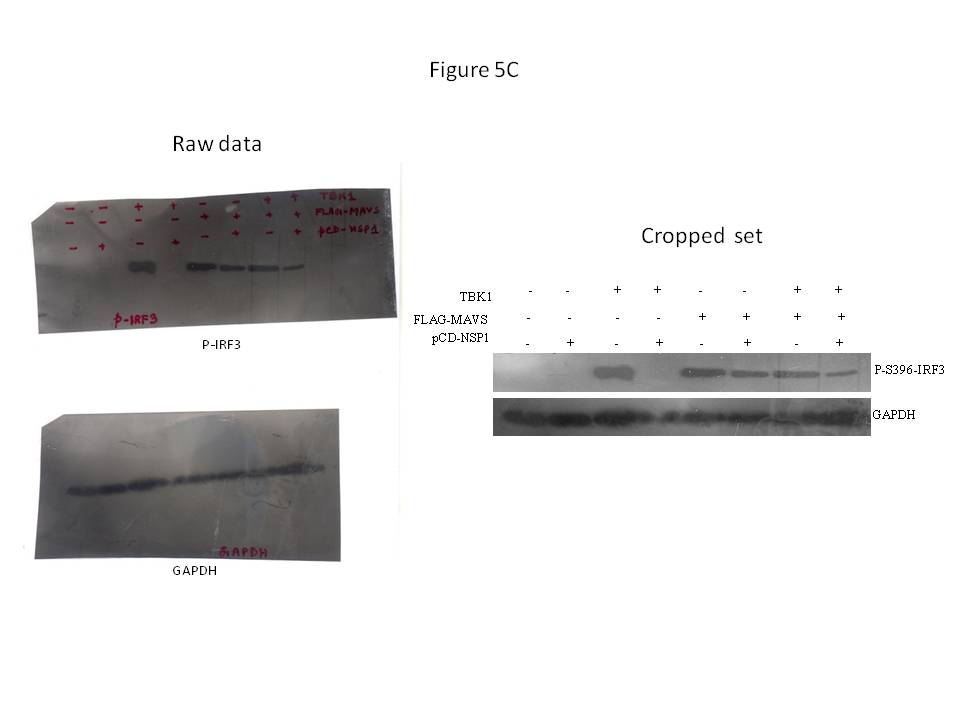

Supplement: S2 File — (ZIP) [file pone.0131956.s003.zip › Fig 5C.JPG]

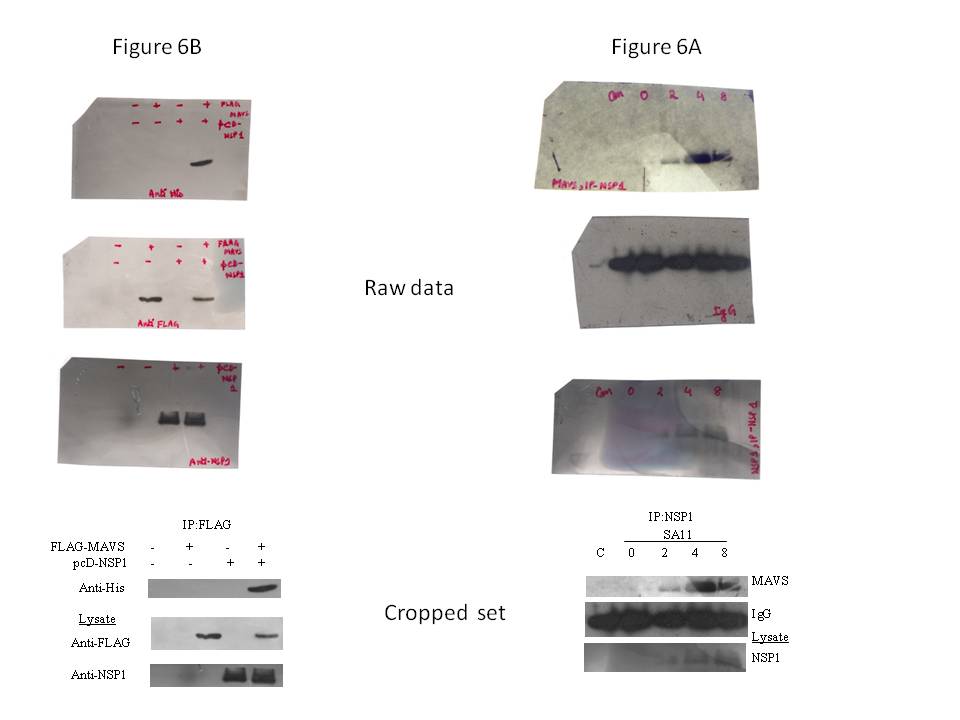

Supplement: S2 File — (ZIP) [file pone.0131956.s003.zip › fig6A,B.JPG]

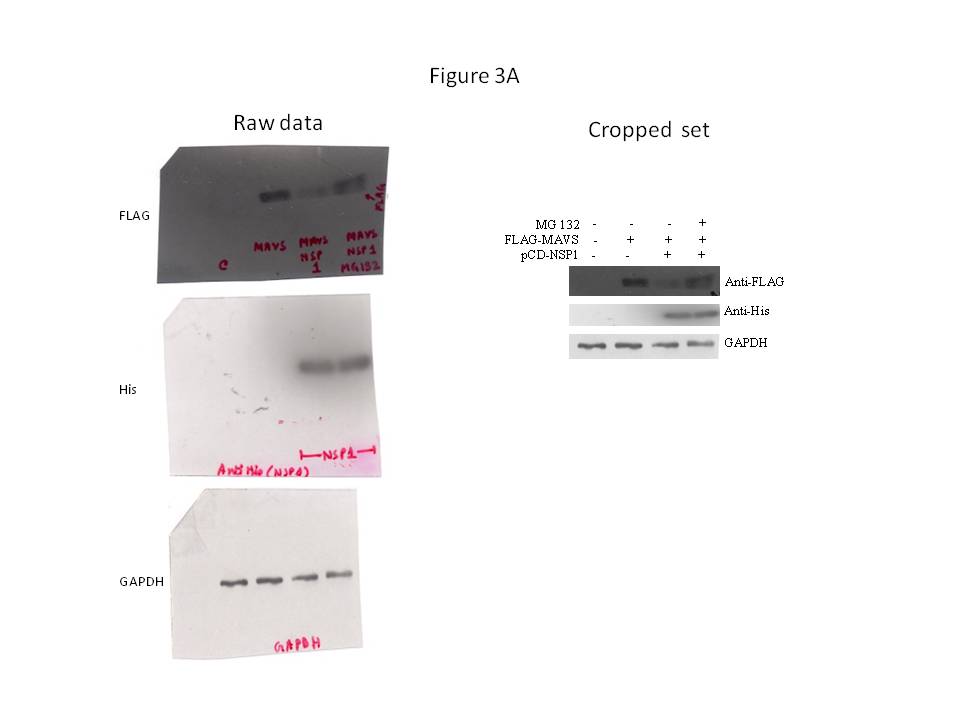

Supplement: S2 File — (ZIP) [file pone.0131956.s003.zip › Fig3A.JPG]

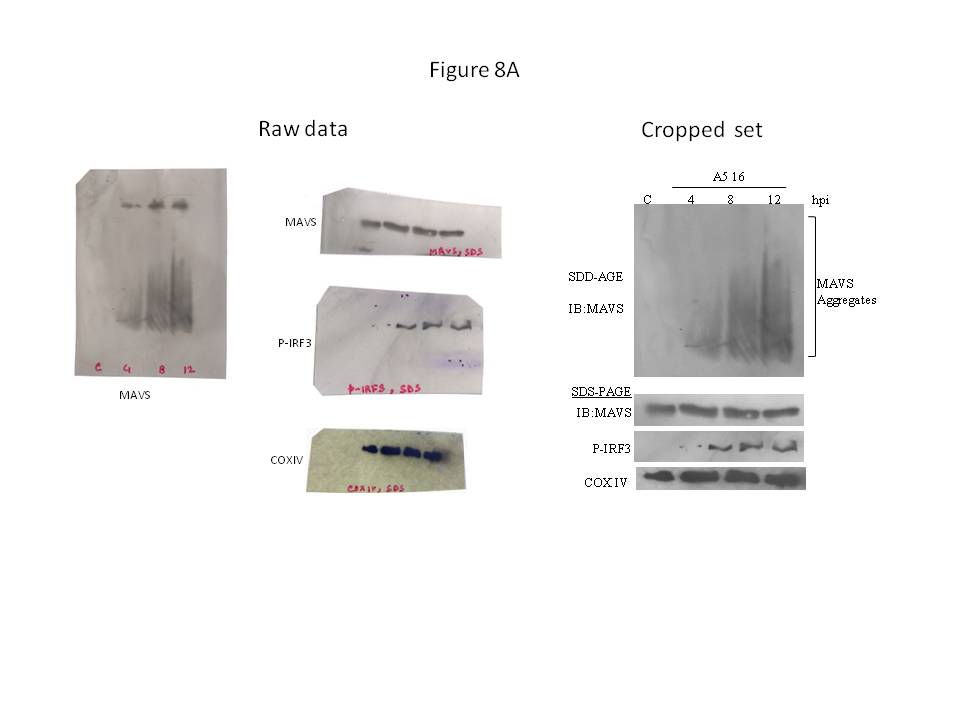

Supplement: S2 File — (ZIP) [file pone.0131956.s003.zip › Fig8A.JPG]

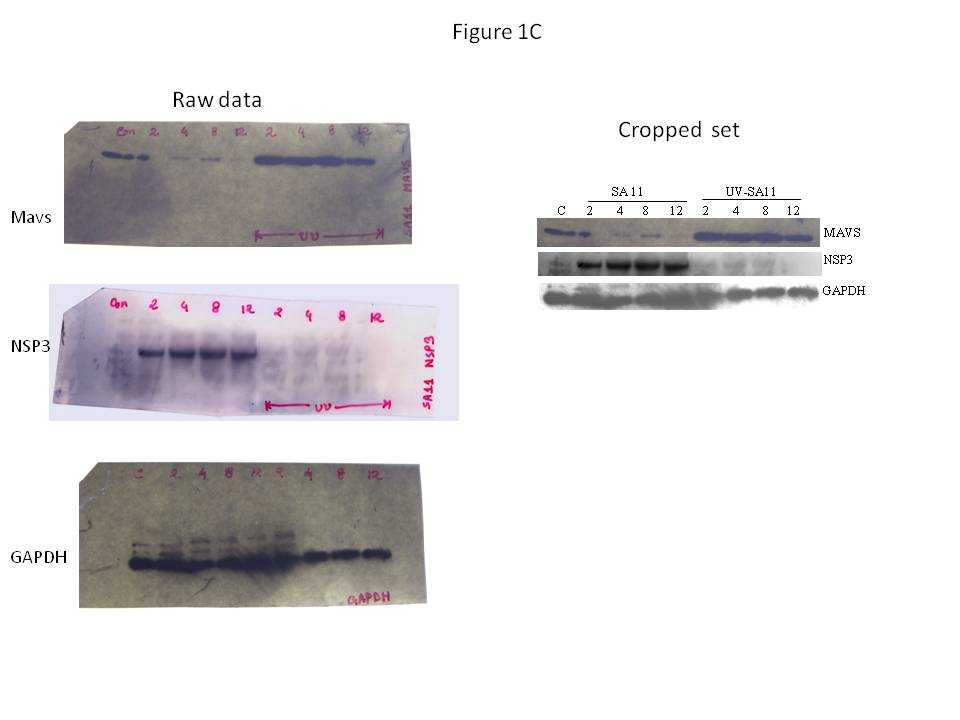

Supplement: S2 File — (ZIP) [file pone.0131956.s003.zip › fig 1C.JPG]
